# Visual Space and Object Space in the Cerebral Cortex of Retinal Disease Patients

**DOI:** 10.1371/journal.pone.0088248

**Published:** 2014-02-05

**Authors:** Elfi Goesaert, Marc Van Baelen, Werner Spileers, Johan Wagemans, Hans P. Op de Beeck

**Affiliations:** 1 Laboratory of Biological Psychology, University of Leuven (KU Leuven), Leuven, Belgium; 2 Laboratory of Experimental Psychology, University of Leuven (KU Leuven), Leuven, Belgium; 3 Division of Ophthalmology, University of Leuven (KU Leuven), Leuven, Belgium; University College of London - Institute of Neurology, United Kingdom

## Abstract

The lower areas of the hierarchically organized visual cortex are strongly retinotopically organized, with strong responses to specific retinotopic stimuli, and no response to other stimuli outside these preferred regions. Higher areas in the ventral occipitotemporal cortex show a weak eccentricity bias, and are mainly sensitive for object category (e.g., faces versus buildings). This study investigated how the mapping of eccentricity and category sensitivity using functional magnetic resonance imaging is affected by a retinal lesion in two very different low vision patients: a patient with a large central scotoma, affecting central input to the retina (juvenile macular degeneration), and a patient where input to the peripheral retina is lost (retinitis pigmentosa). From the retinal degeneration, we can predict specific losses of retinotopic activation. These predictions were confirmed when comparing stimulus activations with a no-stimulus fixation baseline. At the same time, however, seemingly contradictory patterns of activation, unexpected given the retinal degeneration, were observed when different stimulus conditions were directly compared. These unexpected activations were due to position-specific deactivations, indicating the importance of investigating absolute activation (relative to a no-stimulus baseline) rather than relative activation (comparing different stimulus conditions). Data from two controls, with simulated scotomas that matched the lesions in the two patients also showed that retinotopic mapping results could be explained by a combination of activations at the stimulated locations and deactivations at unstimulated locations. Category sensitivity was preserved in the two patients. In sum, when we take into account the full pattern of activations and deactivations elicited in retinotopic cortex and throughout the ventral object vision pathway in low vision patients, the pattern of (de)activation is consistent with the retinal loss.

## Introduction

Retinotopy is the most prominent organizational principle in the visual system. Retinal coordinates are often expressed in terms of a polar coordinate system with the dimensions eccentricity (from central to peripheral positions) and polar angle (from vertical to horizontal offsets and back). Retinotopy is a defining feature of primary and secondary visual areas, hence these areas are often referred to as retinotopic areas [1], [2], [3], [4], [5]. In these areas, the retinotopy is an absolute map: sub-regions in this map respond to stimuli at preferred retinotopic positions and not at all to stimuli at unpreferred locations. However, more and more visual areas have been shown to contain a retinotopic organization [6], [7], [8], [9], [10], and it has been proposed that retinotopy even plays a role as an organizational principle in the highest visual regions. This organization differs from the lower visual areas, as has been demonstrated in [11]. In this study, methods were developed and data were modeled to describe the properties of neuronal population receptive fields (pRF), including receptive field size and the visual field map. The pRF considers a whole collection of neurons with different visual field characteristics, that underlies the activity and visual field properties of a voxel. pRF properties were further compared in primary visual cortex (V1) and a region higher up in the ventral visual stream, lateral occipital (LO). Response differences between the two regions were found that could be explained by a difference in size of the pRF. Data indicate that LO voxels respond to more positions in the visual field than the V1 voxels. The properties of voxels in LO are indicative of the properties of other higher regions of the ventral visual stream.

These higher regions of the visual hierarchy are best known for being organized in terms of category preference, containing face-sensitive, body-sensitive, word-sensitive, and scene-sensitive regions [12], [13], [14], [15], [16]. Nevertheless, a large-scale eccentricity bias has been demonstrated to encompass these category-sensitive regions. According to one proposal [17], [18], [19], this eccentricity bias even precedes and constrains the properties of the category map, so that object categories that are mostly viewed centrally (recognizing faces, reading), are mapped at a different cortical location compared to types of stimuli that are mostly viewed peripherally (scenes, landscapes). However, as the pRF size of the voxels in these regions is much larger, sub-regions typically respond to stimuli presented at many visual field positions. Additionally, this large-scale organization is structured retinotopically only weakly: there seems to be a lack of responses to more paracentral stimuli, which suggests a rough central/eccentric division [20]. In sum, retinotopy is a relevant property throughout the visual system, and a gradual transition is noticed from absolute retinotopic maps to relative retinotopic biases.

This study investigates how the most prominent aspects of these absolute and relative retinotopic maps, and their relationship to maps of category sensitivity, are influenced by the degradation of visual input in humans with retinal defects. Two opposing classes of such defects are, on the one hand, retinal diseases that lead to loss of the foveal or central visual input, such as macular degeneration (MD), and, on the other hand, diseases that degrade predominantly peripheral visual input, such as retinitis pigmentosa (RP). MD can result from inherited conditions with symptoms starting in the first three decades of life (juvenile MD or JMD) and from acquired conditions diagnosed after the fifth decade of life (age-related MD or AMD) [21], [22]. MD is progressive in nature and can produce a central scotoma with a diameter up to 10–20° and severe acuity loss. Most people with macular damage disrupting the fovea will adopt a new more eccentric retinal position as their preferred retinal locus (PRL) for fixation [23]. Retinitis pigmentosa is a hereditary condition with the age of symptom onset ranging from infancy to mid-adulthood. It is characterized by progressive loss of the peripheral visual field leading to tunnel vision (sometimes accompanied by a peripheral island of residual vision) and eventually degradation of central vision [24], [25].

A possible effect of such retinal defects is the induction of neural plasticity, even in the adult brain, as has been demonstrated in animal deafferentation studies [26], [27], [28], [29], and in some human fMRI studies [30], [31]. Nevertheless, the exact nature and size of neural plasticity is still under intensive investigation [32], [33], [34], [35].

There is a more fundamental question, however, that precedes the plasticity discussion: before long-term effects of retinal defects in the visual cortex are considered, the simple lack of input already gives rise to changed activation patterns, and the question is how these changes can best be characterized. This issue is the main focus of this study. The shift in the organization of the visual cortex suggests that the pRF properties of the lower and higher visual areas will be differentially affected by limits in the visual input: as the higher visual areas respond to more positions in the visual field, activity to visible stimuli should be observed across the ventral visual stream, as opposed to being limited to the corresponding locations in the lower visual areas.

We tested two patients, a JMD patient and an RP patient, and we simulated their lesions in two controls.

In the absence of activation for some stimuli, we found that retinotopic maps in the calcarine sulcus sometimes revealed seemingly impossible results: activity for stimuli which the subjects could not see. This unexpected finding was related to the presence of deactivations when stimuli were presented in the visible parts of the visual field. Retinotopy in the higher visual areas was not affected by deactivations, but instead showed a large-scale preference to the visible eccentricity.

Overall, our findings reveal the consequences of visual deprivation upon activity maps in lower and higher visual areas, and indicate the importance of a no-stimulus baseline to detect deactivations, which could influence results in the absence of visual input. As this study considers very simple changes in activation due to reduced visual input, matters of neural plasticity will not be explicitly addressed. However, some implications and the possible relevance of the results to the plasticity question will be discussed.

## Methods

The fMRI study consisted of three experiments: a phase-encoding paradigm to map eccentricity, an object-morph phase-encoding paradigm to characterize object category sensitivity for faces and houses, and a localizer block design experiment to establish regions of interest (ROIs) in the ventral occipitotemporal cortex. The phase-encoding paradigm is adapted from [20], the localizer experiment from [36]. Controls were not tested in the object-morph paradigm, although the second control participated in a previous study [20], where the same paradigm was applied. Both controls participated in the study including the localizer block design, so face- and place-sensitive ROIs were extracted from those data [36].

### Subjects

The first subject was a 28 year old female diagnosed with Stargardt disease, a form of JMD, at the age of thirteen. Her visual acuity was 20/250 in the left and 20/330 in the right eye. Humphrey Field Analyser perimetry with a dot of 0.43° indicated a central scotoma of approximately 24°×18° degrees. The second subject was a 55 year old male diagnosed with retinitis pigmentosa at the age of 32. His visual acuity was 20/40 for both eyes. Goldmann perimetry with a dot of 0.43° indicated a remaining tunnel-shaped visual field of 10°×10° degrees.

The two controls were male, aged 32 and 34 with normal or corrected-to-normal vision. They were both tested with simulated scotomas matching those of the JMD and RP patient. Since both controls were used to test both simulated scotomas, they were not age-matched with the patients. Note that we refrain from making claims about specific differences between controls and a specific patient. The second control also participated in a previous study with similar stimuli [20], and these previously reported eccentricity results are presented as a comparison to the patient data, as an illustration of the appearance of a normal phase-encoding map. All subjects signed an informed consent form and the study was approved by the ethical boards of the Faculty of Psychology and Educational Sciences, and the committee for medical ethics of the KU Leuven (Leuven, Belgium).

### Visual field testing

The visual field was also tested inside the scanner set-up by means of a kinetic perimetry test (mimicking Goldmann perimetry). Visual field testing was done under binocular viewing conditions, since for both subjects preliminary clinical perimetry demonstrated similar scotomas in both eyes. White dots were displayed at maximal luminance against a gray background with a luminance identical to the background used in the retinotopy experiment (stimulus luminance 0.2 log units above background luminance). The visual field was measured with a stimulus diameter of 1.7° (corresponding to Goldmann stimulus size V). The perimetric test consisted of 40 centripetal trials (dots moving inwards from the border to the centre of the screen) followed by 40 centrifugal trials (dots moving in the opposite direction). Dots moved at a speed of 3° per second along 8 different meridians (starting from 0° up to 315° in steps of 45°) each being frequented 5 times in a random order. Patients had to respond when a dot appeared in their visual field (for the RP subject in centripetal trials, for the JMD subject in centrifugal trials) or when it disappeared from their visual field (for the RP subject in centrifugal trials, for the JMD subject in centripetal trials). The mean dot position of centripetal and centrifugal responses together reveals the location of the scotoma border. A scotoma is defined here as the region where dots of stimulus size V at maximal luminance cannot be detected.

For the JMD patient the scotoma measured 20°×13°. For the RP patient, the remaining tunnel-shaped visual field measured 6°×6°. It should be noted that visual field loss is gradual and not an all-or-none phenomenon. Testing with larger dots or a higher stimulus-background contrast could show a smaller scotoma and a larger remaining functional visual field, resulting in some differences in exact scotoma size between visual field testing performed in the scanner compared to the available clinical information mentioned above.

### Stimuli

The eccentricity mapping stimuli were a set of concentric rings with a thickness of roughly 1.6° (JMD and control subjects) or 2° (RP subject). The diameter of the rings varied from 1.5 to 11 cm (resulting in a retinal image size ranging from 2.5° to 18° for the JMD patient plus the control subjects, and from 3° to 23° for the RP patient who was positioned closer to the projection screen). The rings were 24 concentric cut-outs of 8 natural images displaying objects repeated within scenes (sheep, wine bottles, faces, buildings, yoghurt containers, penguins, books and butterflies).

**Figure 1:**
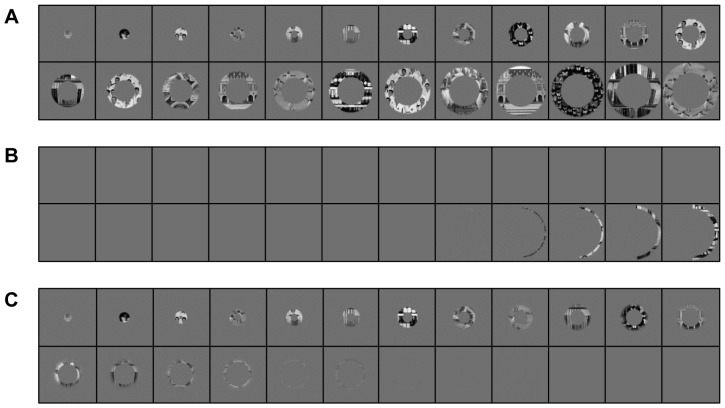
Stimulus set filtered with simulated retinal defects. (A) Example of the stimulus set under normal viewing conditions (B) Stimulus set for the JMD control study. Most conditions show a blank screen, with the five most eccentric stimuli showing part of the rings. (C) Stimulus set for the RP control study. The shift between the visible and not visible stimuli is more gradual, with input present in a large part of the stimulus sequence. Only the most eccentric stimuli are not visible any more.

In the eccentricity mapping paradigm for control subjects, the stimuli (Figure 1A) were filtered with a mean-luminance mask to match the patients' scotomas. In accordance with the perimetric data of the JMD subject obtained inside the scanner set-up, only the five most eccentric rings in the right half of the stimulus display were (at least partly) visible for the JMD control condition (Figure 1B). Since there was no clear evidence for a substantial relative scotoma (i.e., incomplete loss of sensitivity) adjoining central vision loss in the JMD subject, only standard anti-aliasing was applied for the transition between the mask and the visible ring stimuli. For the RP control condition, visual stimulation was preserved in a central tunnel-shaped area with a 6° diameter, consistent with the MRI perimetric results of the RP subject. Adjacent to this tunnel, the visibility of the ring stimuli was gradually decreased towards the periphery in a ring-shaped area with a thickness of 2°. Beyond this annular transition zone, visual stimulation was completely blocked by the mask (Figure 1C). The gradual transition was applied because differences between the results of Goldmann and MRI perimetry pointed towards the presence of a relative scotoma. This was further supported by the RP patient reporting that he occasionally perceived peripheral ‘flashes’ during eccentricity mapping.

The object-morph stimuli consisted of 24 stimuli ranging from 100% face to 100% house, and 22 intermediate morph states, selected on the basis of equal perceptual similarity between subsequent steps (see [20], for details on stimulus construction). There were three versions of the stimulus set available, each one with stimuli at a different orientation angle (45° left, 45° right, front; fMRI results are averaged across the orientation angles). Compared to the study of [20], stimulus size was enlarged in order to maximize visibility and varied for both subjects (12×17°, for the JMD patient, 6×8° for the RP patient).

The localizer stimuli consisted of three types of objects. There were two subtypes of stimuli within each category (old faces, baby faces, hands, torsos, apartment buildings and old houses), with 20 exemplars in each subset (for more information on these stimuli, see [36]). Again, stimulus size was enlarged and differed for both subjects (17°×16° for the JMD patient, 10°×9° for the RP patient).

### Stimulus Presentation

Stimuli were presented on a screen in the scanner by means of a Barco 6400i LCD projector (resolution 1024×768, refresh rate 75 Hz). Since hypersensitivity to light is a frequently observed symptom in retinal disease, a neutral density filter (Lee Filters, optical density  = 0.9) was placed in front of the projector transmitting only 12.5% of the emitted light and hereby avoiding glare in our subjects (mean luminance approximately 104 cd/m^2^). The screen was made visible to subjects by means of a mirror positioned on the head coil. Stimuli were presented using custom software generated in the Matlab environment (Mathworks, Natick, MA) supplemented with PsychToolbox [37].

Stimuli in the eccentricity mapping paradigm were displayed against a gray background with a luminance equal to the mean luminance of the stimuli. In the other experiments a black background was present.

### Fixation Requirements

The RP patient and the two controls fixated a square (0.5°×0.5°) positioned in the centre of the screen during all three stages of the experiment.

The JMD subject used her preferred retinal location (PRL) to fixate the square (1°×1°, enlarged to compensate for the lower visual acuity) located on a screen position required by the stage of the experiment at hand. The fixation square was positioned at the bottom left of the stimulus display in the eccentricity mapping paradigm focusing the central scotoma on the middle of the screen, and positioned on the upper part of the stimuli in the other paradigms to ensure maximum stimulus visibility. The position of the PRL was determined prior to the scan session using customized kinetic perimetry testing under MRI set-up simulated conditions. The PRL was located in the lower left quadrant at a distance of approximately 5° from the scotoma border. The position of the scotoma with respect to the fixation point was very similar in perimetric tests both outside and inside the scanner set-up.

**Figure 2:**
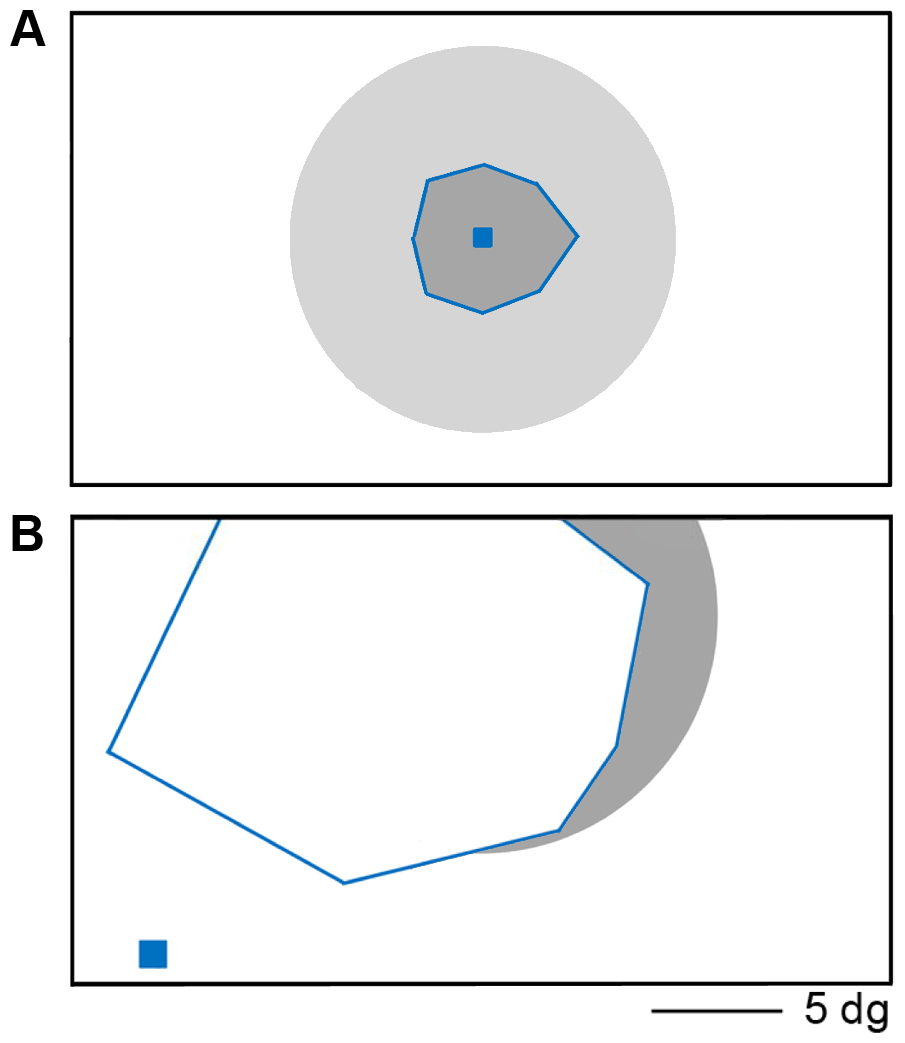
Schematic view of the scanner screen for both patients in the eccentricity mapping paradigm. (A) schematics for the RP patient, with fixation point (blue square) in the centre of the screen. The grey circle represents the extent of the eccentric stimuli, and the blue lines mark the edges of the remaining visual field. Outside these borders the parts of the stimuli that fall outside the remaining visual field are marked in a lighter grey. (B) schematics for the JMD patient. The fixation point is located in the lower left corner, about 5° away from the lower edge of the scotoma. The scotoma (marked with blue lines) covers the centre of the screen, and is slightly larger at the left. The most eccentric stimuli are visible in the right part of the screen (shown in gray).

Figure 2 shows the position of the fixation dot, and the location of the retinal lesions for both RP (Figure 2A), and JMD (Figure 2B) in the scanner during retinotopic mapping. Figure 1 can be considered as an approximation of what part of the stimuli the patients could still see during the experiment (JMD: Figure 1B, RP: Figure 1C). Note, however, that the JMD patient had the rings positioned a bit higher up in the visual field to be able to fit the PRL on the projection screen. There is therefore less input in the lower right quadrant of the visual field for the JMD patient, as can be seen when comparing Figure 1B and Figure 2B. Similarly, for the RP patient the projection screen was positioned closer, causing him to see slightly less of the stimuli than the controls.

### Scanning procedure

#### Experimental setup

The fMRI data were collected for each patient during two scan sessions. The JMD and RP patient completed 8 eccentricity runs, both patients completed 8 object-morph and 6 localizer runs. The two controls completed 8 eccentricity runs for each simulated scotoma (JMD and RP). The localizer data were collected in a previous experiment (see [36] for details), and for the second control object-morph data and eccentrity data without a simulated scotoma were available as well (see [20] for details).

Eccentricity and object-morph runs were designed to be analysed with phase-encoding Fourier analysis [20], and contained 6 repetitions of an eccentric or object-morph sequence (rings expanding or contracting, or stimuli morphing from face to house or house to face, with one sequence lasting 24 s, resulting in a frequency of 1/12 Hz). Stimuli were presented for 500 ms, followed by a short fixation period of 500 ms. A longer fixation period was presented at the start and the end of each run (14 s), which was used as the no-stimulus fixation baseline that was implicitly modelled in a GLM model.

In each eccentricity run, the content of the rings was randomly chosen from the 8 natural image stimuli, given the following restrictions: no two subsequent rings showed the same stimulus and each stimulus appeared 18 times per run. In each object-morph run the stimulus orientation angle was fixed (either 45° left, 45° right or frontal orientation), meaning that in each run only one specific face/house orientation was presented.

The localizer experiment was a block design in which each run consisted of 6 stimulus blocks (one for each of the 6 object subcategories present in the stimuli) and three fixation periods (at the beginning, middle and end of the run). Each block lasted for 15 s. In each stimulus block the 20 corresponding stimuli were presented in a random order. Stimulus presentation time was 0.5 s with an interstimulus-interval of 250 ms. The order of the stimulus blocks was randomized, and counterbalanced across the two patients.

The three different parts of the study required different tasks from the subjects. During eccentricity mapping, all subjects were asked to detect the stimulus with the lowest luminance (butterfly stimulus). The task of the second control in the eccentricity mapping paradigm without a simulated scotoma was to detect a color change. For the RP patient, additional data were collected for this experiment in which the patient had to detect a change in the luminance of the fixation dot. In the Results section, only the results for the runs with the stimulus task will be shown, the results for the fixation dot task can be found in the Supporting Information section (Figure S1). During the object morph-runs, both the JMD and RP patients had to indicate a downward change of mean luminance which occurred three times per stimulus sequence (18 times in total per run). This was designed to match the type of task used in the original object-morph experiment [20], where changes in color had to be detected. In the localizer runs, both patients were asked to perform a 1-back task requiring a response when a stimulus was repeated immediately.

#### Data acquisition

The functional imaging data were collected on a 3T Philips Intera magnet (Department of Radiology, KU Leuven, Leuven, Belgium). It has an 8-channel SENSE head coil with an echo-planar imaging sequence (86 time points per time series or “run” for all phase-encoding paradigms; repetition time, 2000 ms; echo time, 29.8 ms; acquisition matrix 104×104 resulting in a 2.0 by 2.0 mm^2^ in-plane voxels size and 33/32 slices for the patients and the controls, respectively) oriented approximately halfway between a coronal and horizontal orientation and including most of cortex except the most superior parts of frontal and parietal cortex, with slice thickness 2 mm and interslice gap 0.2 mm. For the localizer block paradigm, settings were similar but 75 time points were collected per run with a repetition time of 3000 ms and 48 slices for the patients. A T1-weighted anatomical image (resolution 0.98 by 0.98 by 1.2 mm; 9.6 ms TR, 4.6 ms TE, 256×256 acquisition matrix, 182 coronal slices) was also acquired.

### Data analysis and visualization

Before the actual statistical analyses, all fMRI data were pre-processed using SPM 5 (Wellcome Department of Cognitive Neurology, London). First, the functional images were corrected for differences in acquisition time and realigned to correct for head movements. Functional images were then co-registered with their anatomical image and the co-registered anatomical image was segmented. Subsequently, all images were spatially normalized to MNI space (resampling to voxel size 2×2×2 mm) using the parameters resulting from the segmentation step. Finally, functional images were smoothed with a 4 mm full-width at half maximum Gaussian kernel.

A Fourier analysis was performed on the data from the eccentricity and object-morph runs (see [20], for more details). For each voxel a mean value was calculated for similar runs (expanding, contracting, face to house or house to face). For each of these conditions, the runs were normalized to a mean of zero, a temporal drift correction was applied, and subsequently a Fourier analysis followed extracting the amplitude and phase corresponding to the stimulus frequency (assuming a hemodynamic delay of 6 s). The opposite runs got their phase signs reversed, and were subsequently unwrapped and averaged across runs. This resulted in a single phase value for each voxel, which was transformed to a scale ranging from 0 to 2*pi. The range of this scale represents phase values which correspond to preferences to the phase-encoding stimuli. The lower end of the scale, with values approximating 0, reflects preferences to the central stimuli or face-like stimuli, while the higher values, close to 2*pi or 6.28, reflect preferences to the peripheral stimuli or the house-like stimuli. In the figures, these values were color-coded and the values for each of the voxels were mapped onto the inflated cortex. The amplitude which corresponded to the stimulus frequency was normalized by dividing it with the total Fourier power (excluding the lowest frequencies and harmonics). A similar analysis on white matter and outside brain-voxels was performed to establish a statistical significance threshold for the amplitude of the sinusoidal modulations (p<0.05 uncorrected). Phase responses are only shown for voxels that passed this threshold. For JMD and RP the thresholds were similar, with an amplitude value of 0.45 and 0.40, respectively, for the eccentricity mapping and 0.33 (JMD) and 0.38 (RP) for the object-morph paradigm. The controls had thresholds that were established for the eccentricity mapping for each simulated scotoma (control 1: 0.36 for the JMD scotoma and 0.34 for the RP scotoma; control 2: 0.39 for the JMD scotoma and 0.37 for the RP scotoma). The eccentricity mapping data for the second control without a simulated scotoma were collected in a different study [20] and were analyzed without filtering out the low spatial frequencies. For that reason, the threshold was lower at 0.17.

Additionally, the data of the phase-encoding eccentricity mapping method were re-analyzed as a standard block design by dividing the 24 retinotopic stimuli in three conditions: central, paracentral and peripheral. Each condition consisted of 8 stimuli. A number of contrasts were constructed, with each condition contrasted against baseline (the 14s fixation blocks at the beginning and the end of the run). Similarly, the object-morph phase-encoding data could be divided into face-like stimuli, house-like stimuli and morphs, with 8 stimuli in each condition, and contrasts could be extracted comparing the face-like and house-like conditions to baseline. From this design, a GLM model was constructed where beta values could be extracted for each condition and each run per voxel. The mean beta values across sessions were calculated for specific regions of interest (see next section).

Aside from whole-volume analyses, additional analyses focused upon a number of ROIs. ROIs in the ventral visual stream were selected from the block-design localizer experiment by contrasting the face conditions with the house and body parts conditions (for the face-sensitive areas, FA, containing the fusiform face area and nearby regions), and contrasting the house conditions with the face and body parts conditions (for the house/place-sensitive areas, PA, containing the parahippocampal place area). The localizer contrasts were thresholded at p<0.0001 uncorrected. All FAs and PAs at this threshold were selected if they were positioned in the lower part of the ventral visual cortex. Control 2 had one patch more anterior in the ventral stream, but this was not selected due to the large deviation of its location to the FAs of the patients and the other control.

To illustrate the time course of the activation during the eccentricity mapping, the time course of a stimulus sequence was averaged across runs and across all voxels in a specific ROI. Runs with opposing stimulus sequences (central to periphery and periphery to central) were averaged after reversing the pattern of one of the two sequences. This results in a time series of responses (12 data points in total). The scale of the time course activity was transformed to the range of the average beta values in the region of interest, to match the pattern to the activation and deactivation responses that were extracted from the GLM model. This was done by multiplying the values of the time course with the range of the mean beta values, and dividing by the range of the old scale. In a next step, the difference between both scales was calculated, and added to the values of the time course, shifting the scale along the Y axis. This results in a scale where the range corresponds to the range of the beta values in the ROI, and has a maximum and minimum that corresponds to the maximum and minimum beta value of the ROI.

All data were visualized using CARET software [38]. The anatomical image was normalized and resampled to 1×1×1 mm, segmented, and the cortical surface was reconstructed and inflated to clearly show the ROIs and the phase-encoded pattern. This software was also used to manually select two regions in the calcarine sulcus for each subject. The ROIs where selected to illustrate the positive and negative BOLD activations, at threshold p<0.05 uncorrected.

## Results

### Eccentricity mapping in lower visual areas

#### Eccentricity mapping under normal viewing conditions

**Figure 3:**
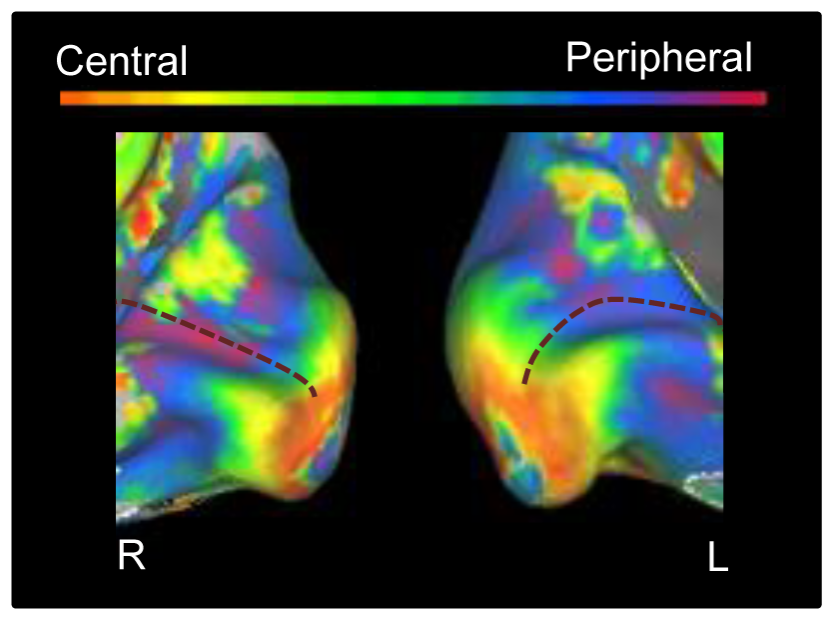
Relative preference for different eccentricities in lower visual areas without a simulated scotoma. The medial view of the posterior part of right and left hemisphere is shown on an inflated cortical surface for control 2. The approximate location of the calcarine sulcus is marked with a dotted line. The color legend is shown above (orange-red for central stimuli, green for paracentral stimuli, blue-purple for peripheral stimuli).

Control 2 was tested in a previous study with the same design and stimuli, but without a simulated scotoma. This provides a good baseline of the characteristics of a normal retinotopic map, which is important information before we compare this normal situation to the new data in patients and controls with simulated scotoma. Figure 3 shows this normal pattern for the right and left hemisphere, around the calcarine sulcus. Note the expected gradual transition from central preferences in the more posterior part of the calcarine sulcus, to more peripheral when moving more anterior in the calcarine sulcus, spanning the entire range of preferences to all eccentricity positions.

#### JMD patient

**Figure 4:**
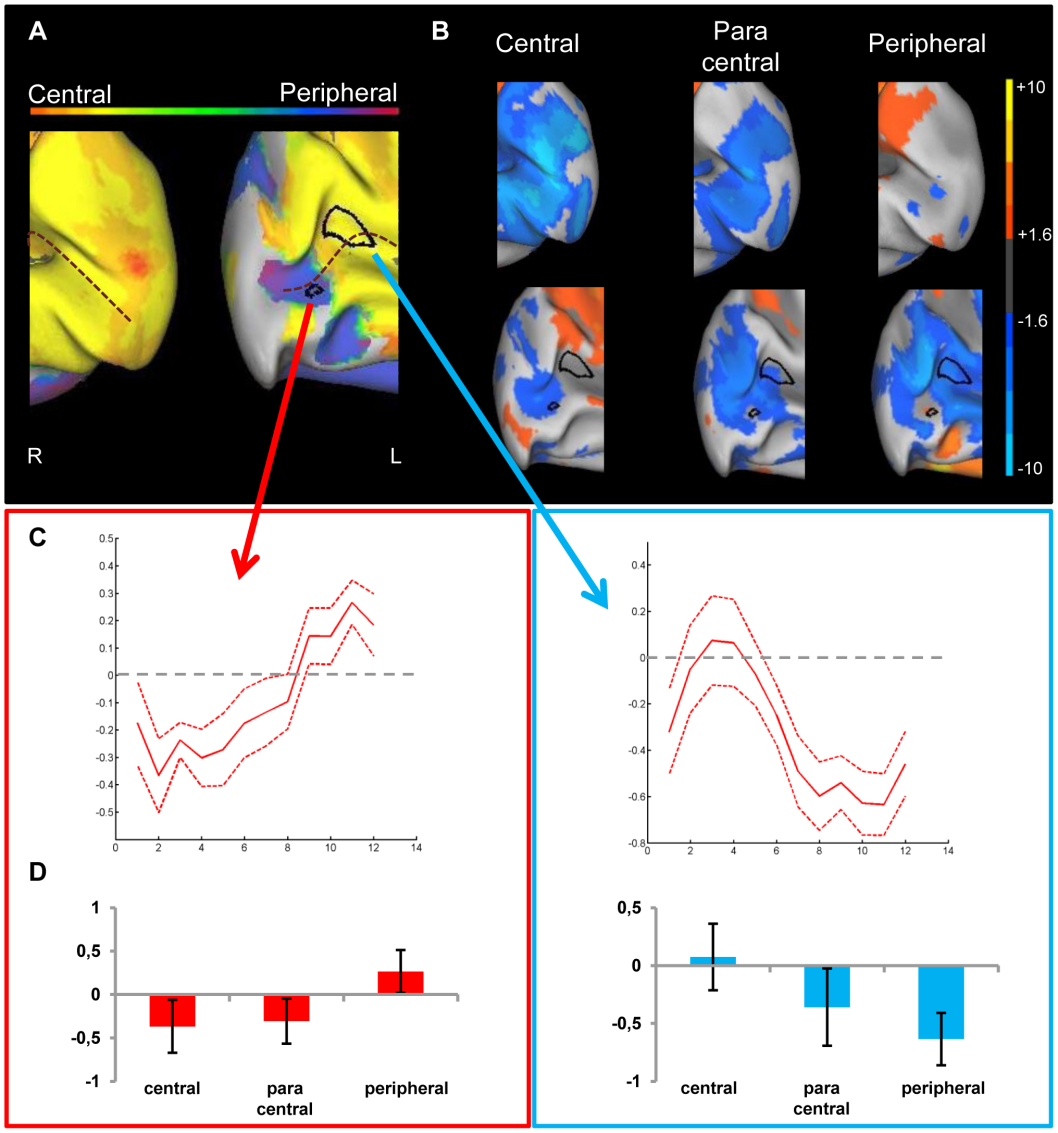
Preference and activity patterns for different eccentricities in lower visual areas for the JMD patient. (A) The medial view of the posterior part of right and left hemisphere is shown on an inflated cortical surface. The approximate location of the calcarine sulcus is marked with a dotted line. The color legend is shown above (orange-red for central stimuli, green for paracentral stimuli, blue-purple for peripheral stimuli) and reflects the relative preference to the different eccentricities. In black two regions are marked which are further characterized for illustration purposes. The data of one region (red arrow/box) are mostly dominated by a positive response, and for the other region (blue arrow/box) mostly by a negative response compared to a no-stimulus baseline (B) Activity patterns in both hemispheres compared to a fixation baseline, at p<0.05 uncorrected for one of three conditions: central (8 most central stimuli, contrasted against baseline), paracentral (8 paracentral stimuli, contrasted against baseline) and peripheral (8 most eccentric stimuli, contrasted against baseline). The selected ROIs now show the underlying positive and negative responses. (C) Time course averaged across runs and across stimulus sequences to represent the response in a selected ROI to different eccentricities. The red dotted lines represent the 95% confidence intervals (calculated using the variation across runs). (C, left panel) ROI with a small positive response to peripheral stimuli compared to a fixation baseline and negative responses to the other conditions (C, right panel) ROI with a close to zero response to central stimuli and negative responses to the other conditions.(D) Average beta values in each selected ROI. The left panel indicates activity of a ROI that shows a small positive response to the (visible) peripheral stimuli, while the right panel shows a ROI with negative responses to unstimulated parts of the visual field and the absence of activation in the central condition.

The results for the retinotopic phase-encoding data in early visual cortex are shown in Figure 4A, in which a medial view of both hemispheres on an inflated cortex can be seen. Main area of interest is the calcarine sulcus. This map has very different characteristics when compared with the normal pattern shown previously. The right hemisphere in the patient reveals a massive dominance for central or near-central stimuli, despite the large lesion of this patient spanning all of the foveal retina. In the left hemisphere, this preference is visible as well, although some responsiveness to peripheral stimuli can be seen there too. The central preference spans the whole of the calcarine sulcus, indicating that even voxels in the lesion projection zone (LPZ) seem to show a preference for central stimulation.

A comparison of visual responses with a no-stimulus fixation baseline reveals that all seemingly impossible responses to insensible stimuli are due to massive deactivations for the contrasted perceivable stimuli. This is demonstrated in Figure 4B, which shows for both hemispheres the activation and deactivation patterns compared to baseline in the calcarine sulcus when the fMRI data are analyzed as a block design. There are massive deactivations present in the calcarine sulcus when peripheral stimuli are presented (p<0.05 uncorrected; for higher thresholds, see Supporting Information, Figure S2A and S2B). When central stimuli are shown, there is a lack of activation. In a phase-encoding analysis, all responses are compared with each other, with the no-response coming out as much stronger than the negative responses. The result is a preference for conditions in which there is no actual response present.

The JMD patient has little or no positive activations in V1. Only in the left hemisphere, a small patch of activation to the peripheral stimulus in the anterior part of the calcarine sulcus can be found. This is probably caused by the large scotoma of the subject: the diameter of the scotoma closely matches the actual size of the largest stimuli on the screen. This reduced visibility of the stimulus is demonstrated by the results to the task performed by the JMD patient: the average performance was 28% correct, with no responses to the central and paracentral stimuli.

The positive and negative responses are further explored in Figure 4C and Figure 4D. A region of interest was drawn around the small positive response in the middle of the calcarine, and another patch containing a large negative response in the peripheral vs. baseline condition was selected more anteriorly in the calcarine sulcus. We show the time course for each region (Figure 4C, left panel for the positive response and Figure 4C, right panel for the negative response). Additionally, the mean beta values to each of the conditions when the data are analysed as a block design (central vs. baseline, paracentral vs. baseline, peripheral vs. baseline) are shown in Figure 4D. The time course in Figure 4C, left panel shows a negative value for central stimuli that turns positive only in the more peripheral conditions, where stimuli were still visible to the patient. Figure 4C, right panel starts with a value closer to zero in the central condition (mean beta value close to zero in Figure 4D, right panel), and the more peripheral stimulation is associated with a strongly negative value. While both time courses show negative values, the difference of its effect on the phase-encoding pattern lies in the non-negative value they are being compared with: Figure 4C, left panel and 4D, left panel have negative values being compared to a positive response, resulting in a phase preference to a stimulus which is visible to the patient. Figure 4C, right panel and 4D, right panel demonstrate a negative value being compared with a zero response, which results in a preference to a stimulus which was not perceived by the patient.

#### JMD controls

**Figure 5:**
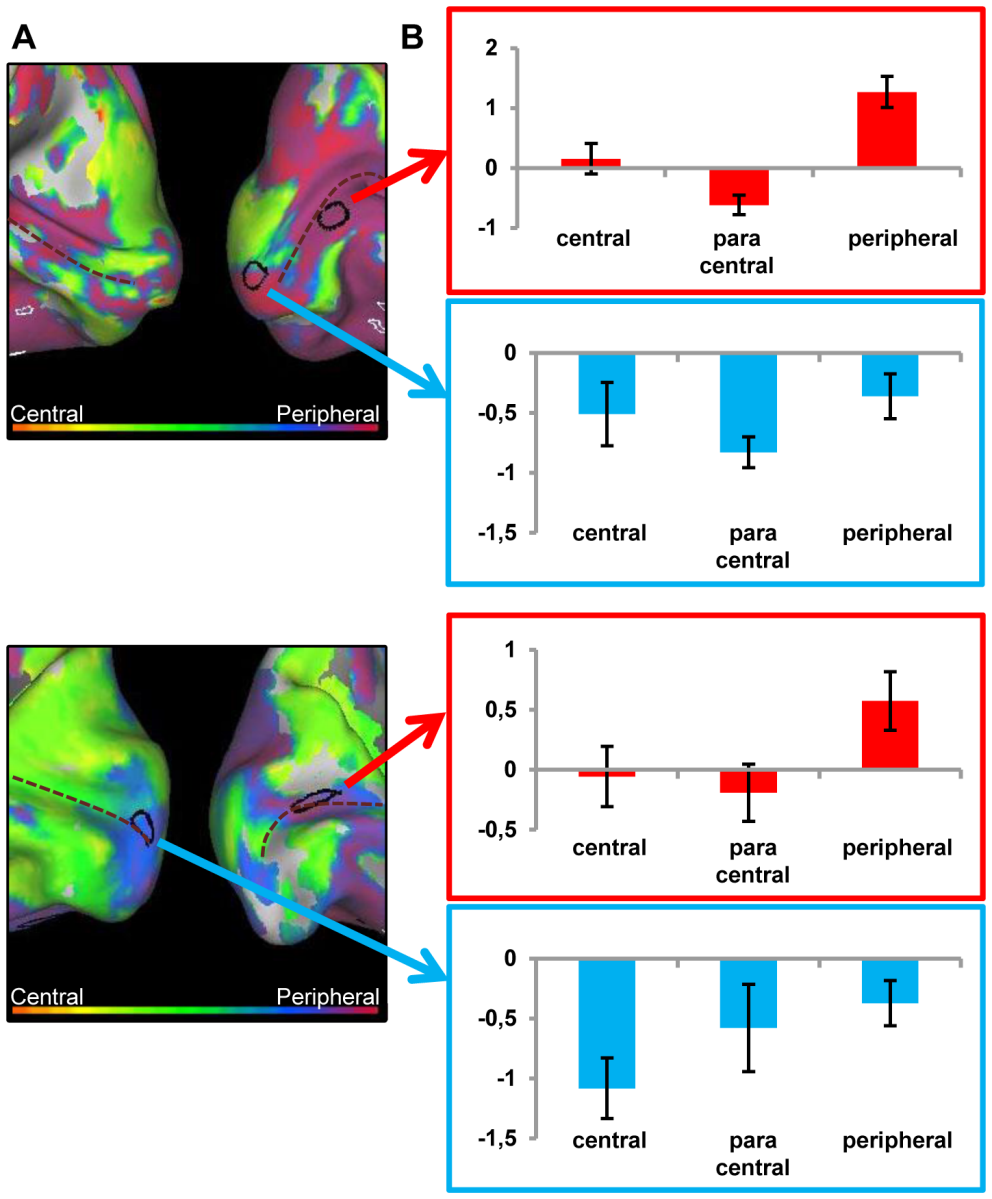
Preference and activity patterns for different eccentricities in lower visual areas for the JMD controls. (A) The medial view of the posterior part of right and left hemisphere is shown on an inflated cortical surface for the two controls (control 1: upper figure, control 2: lower figure). They were tested with the stimulus set simulating the JMD scotoma. The approximate location of the calcarine sulcus is marked with a dotted line. The color legend is shown above (orange-red for central stimuli, green for paracentral stimuli, blue-purple for peripheral stimuli) and reflects the relative preference to the different eccentricities. In black two regions are marked which are further characterized for illustration purposes. The data of one region (red arrow/box) are mostly dominated by a positive response, and for the other region (blue arrow/box) mostly by a negative response compared to a no-stimulus baseline. (B) Average beta values in each selected ROI. The red arrows and box indicate activity of a ROI that shows a positive response to the (visible) peripheral stimuli, while the blue arrows and box show a ROI where negative responses to unstimulated central parts of the visual field cause a phase preference in the absence of activation in the other conditions.

Figure 5A shows the phase-encoding data in the lower visual areas of both controls for which the stimuli were filtered to match the retinal defect in the patient (top part of Figure 5A: control 1; bottom part of Figure 5A: control 2). The average beta values represent the activity pattern compared with baseline in Figure 5B when the data are treated as a block design. Different from the JMD patient, the dominant phase preference in both controls is paracentral/peripheral, rather than (para)central. In particular, spots with a definite peripheral preference can be seen in the more posterior part of the calcarine sulcus, where a preference to more centrally presented stimuli is expected (Figure 5A). These spots are further investigated by selecting a region of interest, and comparing them with peripherally preferential spots more anterior in the calcarine sulcus. The more posterior spots all show deactivation, while the more anterior spots have a stronger positive response to the peripheral condition (Figure 5B). While it seems the posterior spots show no no-activation condition as with the JMD patient, there seems to be a mix of zero-responses and negative values in the peripheral condition (see Supporting Information, Figure S3, and Figure S2C, S2D and S2E, S2F for activation patterns at higher thresholds). The dominance of the phase value in this ROI is again caused by comparing conditions of which none has a positive response, with the peripheral condition having the least negative response of the three conditions, and coming out as dominant. The more anterior spots show a positive response in the peripheral condition, resulting in a phase preference to the visible stimuli.

#### RP patient

**Figure 6:**
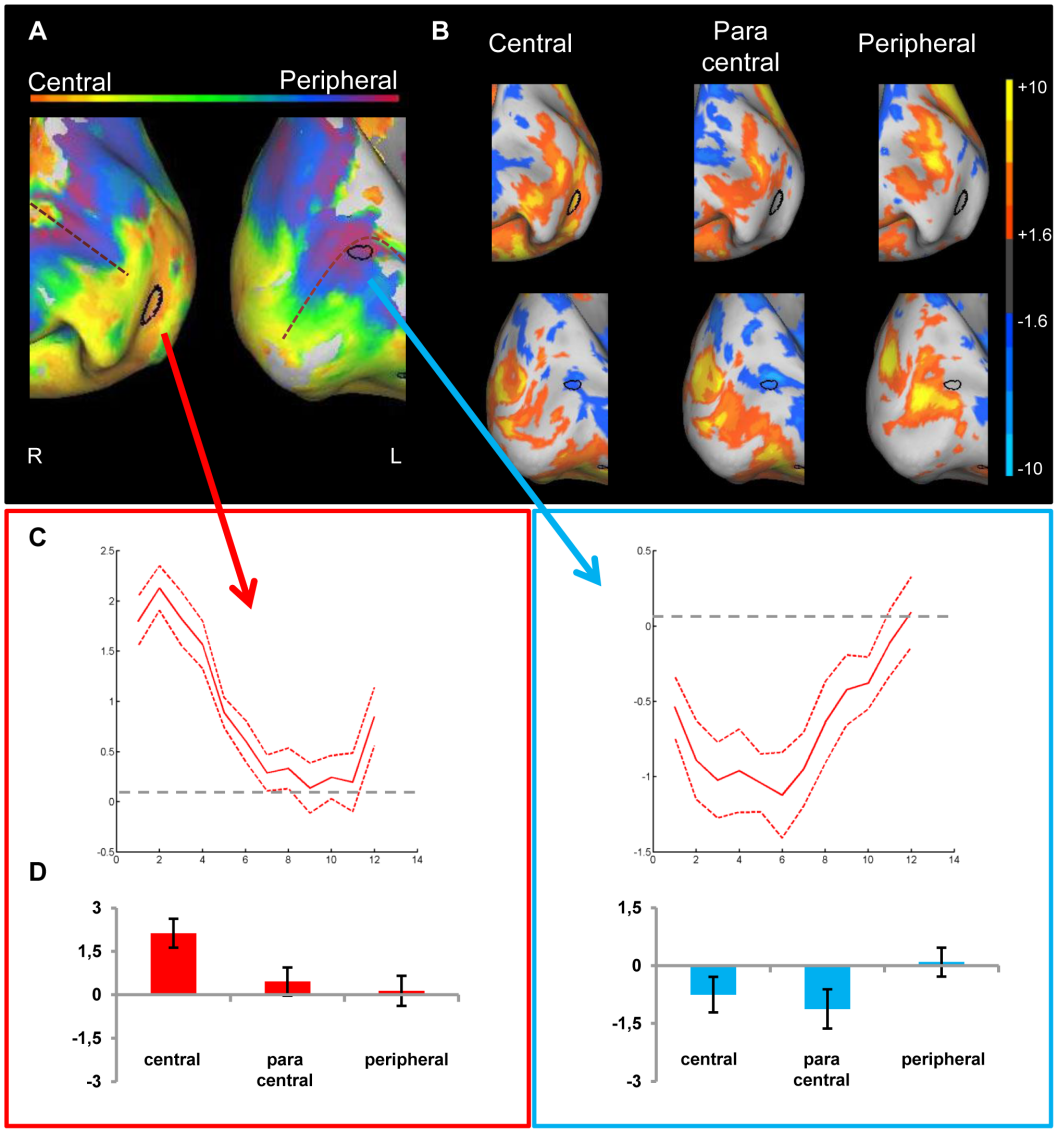
Preference and activity patterns for different eccentricities in lower visual areas for the RP patient. (A) The medial view of the posterior part of right and left hemisphere is shown on an inflated cortical surface. The approximate location of the calcarine sulcus is marked with a dotted line. The color legend is shown above (orange-red for central stimuli, green for paracentral stimuli, blue-purple for peripheral stimuli) and reflects the relative preference to the different eccentricities. In black two regions are marked which are further characterized for illustration purposes. The data of one region (red arrow/box) are mostly dominated by a positive response, and for the other region (blue arrow/box) mostly by a negative response compared to a no-stimulus baseline (B) Activity patterns in both hemispheres compared to a fixation baseline, at p<0.05 uncorrected for one of three conditions: central (8 most central stimuli, contrasted against baseline), paracentral (8 paracentral stimuli, contrasted against baseline) and peripheral (8 most eccentric stimuli, contrasted against baseline). The selected ROIs now show the underlying positive and negative responses. (C) Time course averaged across runs and across stimulus sequences to represent the response in a selected ROI to different eccentricities. The red dotted lines represent the 95% confidence intervals (calculated using the variation across runs). (C, left panel) A positive response to the most central stimuli, with a strong drop in activation to a near zero response when more eccentric stimuli are presented (C, right panel) Strong deactivations for the central and paracentral stimuli, and a response close to zero for the peripheral stimuli (D) Average beta values in each selected ROI. (D, left panel) Positive responses in the central and paracentral conditions, and a near zero response in the peripheral condition. (D, right panel) Negative responses (beta values) for the (para)central conditions and a near zero response to the peripheral condition.

Figure 6A displays the results for the RP subject around the calcarine sulcus. The phase-encoding data show a seemingly normal eccentricity map that spans the complete color scale (as one expects to see in a control subject with normal vision, see Figure 3 and [20] for more examples) and spans the entire calcarine sulcus, well into the LPZ. Even though at least part of the tested peripheral field is affected by the retinal defect, a preference is found even for the most eccentric rings.

As in the JMD patient, deactivation patterns again provide at least a partial explanation: a relatively extended deactivation (versus the fixation baseline) is found anteriorly around the calcarine sulcus especially when central or paracentral stimuli are presented (Figure 6B, see Supporting Figure S4A and S4B for activation at higher thresholds). The most anterior part of these deactivated regions is not activated by peripheral stimuli (more posterior regions are), but nevertheless the phase-encoding analyses suggest responses to peripheral stimuli in this most anterior region because the no-activation by peripheral stimuli is higher than the deactivation by central stimuli.

This effect of the deactivations is not very widespread in this patient. In fact, even when focusing solely upon the positive activation patterns (compared to fixation), a strong pattern of activation for the RP patient can be seen even for the most eccentric stimuli. This finding stands in contrast to the results from the perimetry test. However, as mentioned before, lack of functionality according to a perimetry test does not necessarily indicate absolute absence of input. The RP patient had an average score of 25%, indicating that not all stimuli were visible (compared to 98% correct in the object-morph task). When examining the stimuli the RP patient responded to, he only did so for the first 10 stimuli in the sequence at most, again showing that the most peripheral stimuli were not visible. However, during the debriefing of the experiment the RP patient reported that occasionally he noticed some flashes in the periphery. This is an indication that there is a gradual decrease in sensitivity (a relative scotoma) beyond the preserved visual field that was defined with the official perimetry test.

#### RP controls

**Figure 7:**
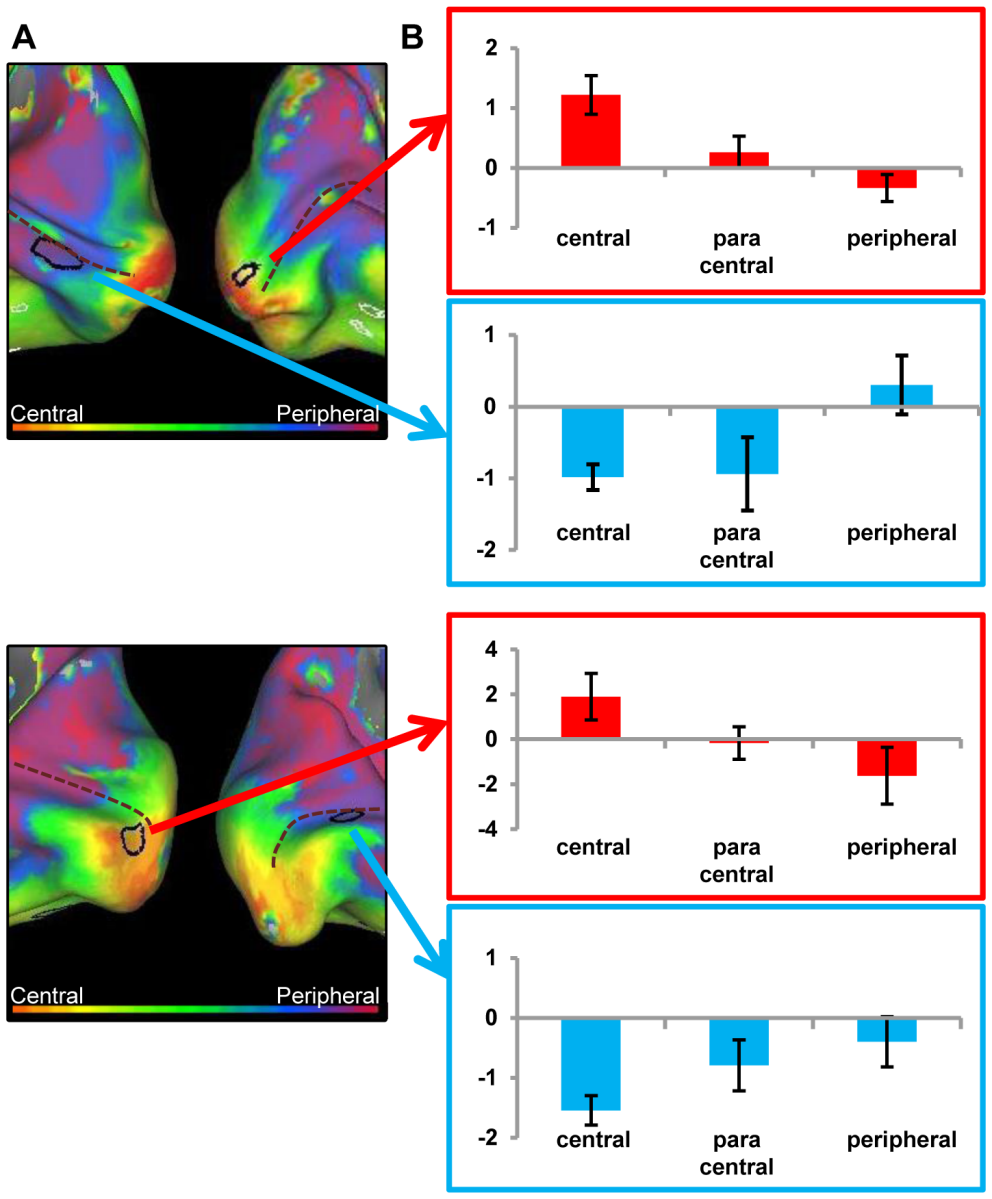
Preference and activity patterns for different eccentricities in lower visual areas for the RP controls. (A) The medial view of the posterior part of right and left hemisphere is shown on an inflated cortical surface for the two controls (control 1: upper figure, control 2: lower figure). They were tested with the stimulus set simulating the RP scotoma. The approximate location of the calcarine sulcus is marked with a dotted line. The color legend is shown above (orange-red for central stimuli, green for paracentral stimuli, blue-purple for peripheral stimuli) and reflects the relative preference to the different eccentricities. In black two regions are marked which are further characterized for illustration purposes. The data of one region (red arrow/box) are mostly dominated by a positive response and for the other region (blue arrow/box) mostly by a negative response compared to a no-stimulus baseline (B) Average beta values in each selected ROI. The red arrows and box indicate activity of a ROI that shows a positive response to the (visible) central stimuli, while the blue arrows and box show a ROI where negative responses to unstimulated peripheral parts of the visual field cause a phase preference in the absence of activation in the other conditions.

The pattern of the RP controls is very similar to the RP phase-encoding pattern, as can be seen in Figure 7A (upper half: control 1; lower half: control 2). Again, in spite of the lack of input in the peripheral condition, a seemingly normal retinotopic map can be seen spanning the calcarine sulcus. The selected ROIs demonstrate some of the effects underlying this phase pattern, and they are investigated in more detail in Figure 7B (and see Supporting Information, Figure S5 for a visual representation of activation patterns in the calcarine sulcus of the controls). As with the RP patient, the responses in the more anterior part of the calcarine sulcus can be explained in terms of strong deactivations in the visible conditions (central and paracentral) that are compared to a peripheral condition where responses were less negative (control 2, lower part Figure 7B and Supporting Figure S5B) or close to zero (control 1, upper part Figure 7B and Supporting Figure S5A). In the more posterior selected ROIs, the phase preference to central stimuli is caused by a strong positive response, regardless whether the other conditions show deactivation (control 2, lower part Figure 7B and Supporting Figure S5B) or lower/no activation (control 1, upper part Figure 7B and Supporting Figure S5A).

### Eccentricity mapping in higher visual areas

#### JMD patient and controls

**Figure 8:**
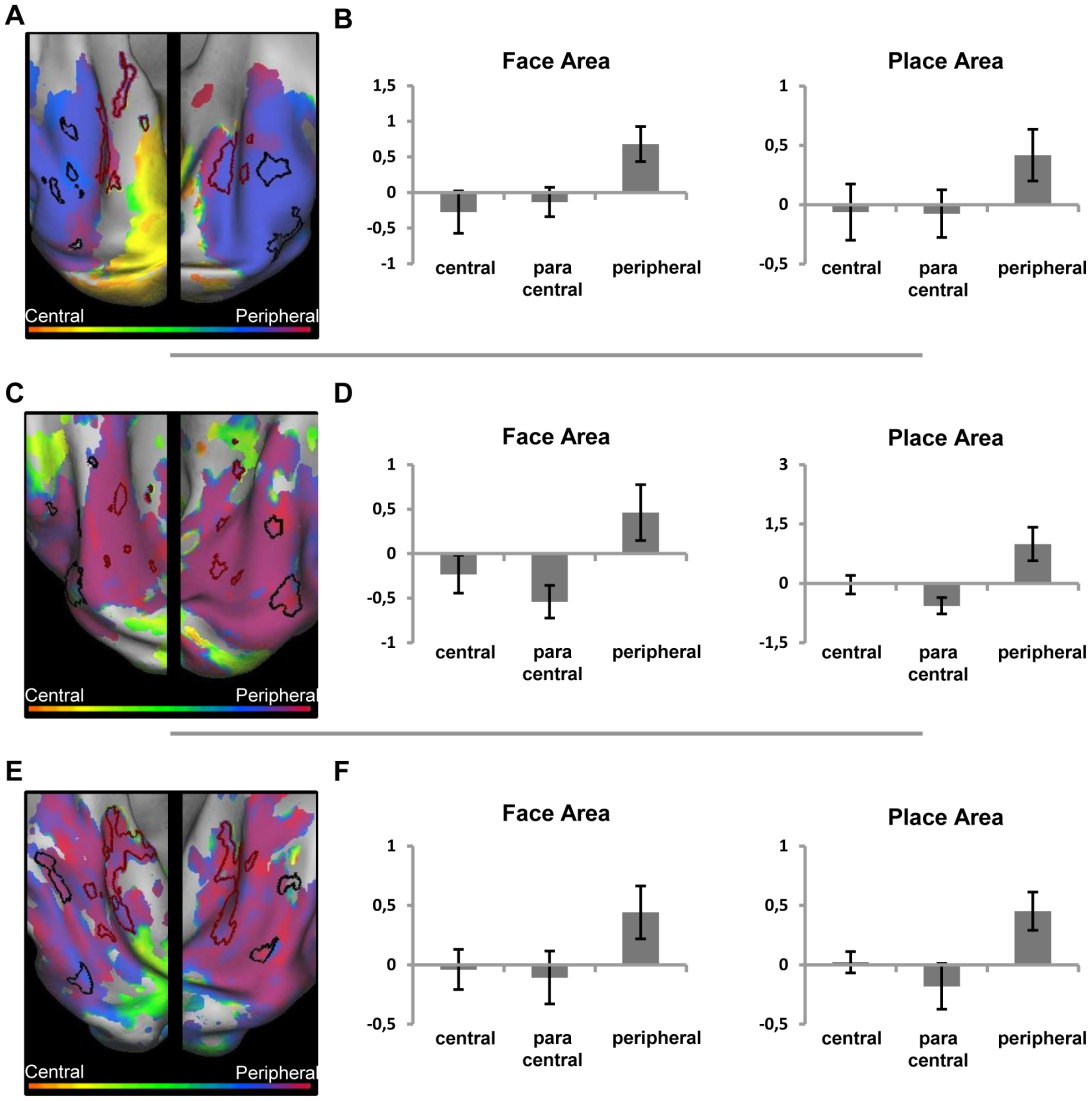
Preference and activity patterns for different eccentricities in the ventral cortex with central (simulated) scotoma. (Left) Relative preference in the eccentricity mapping paradigm for the JMD patient (A), control 1 (C) and control 2 (E), shown on an inflated hemisphere. The color legend is shown above (orange-red for central stimuli, green for paracentral stimuli, blue-purple for peripheral stimuli). The black lines mark the face-sensitive areas (FA), the red lines mark the house (place)-sensitive areas (PA) defined by the blocked localizer design. (Right) average beta values of three conditions, when the eccentricity data are analyzed as a block design and compared to a fixation baseline, in both the FA and PA region for the JMD patient (B), control 1 (D) and control 2 (F).

The map of the eccentricity-related responses in high-level visual cortex, does not reveal the division between periphery/fovea as found by [17], [19], [20]. Instead, the ventral visual cortex of the JMD patient shows a strong preference for peripheral stimuli (Figure 8A). Figure 8B shows the average activation in both the FA and PA. While there are deactivations in the central and paracentral conditions, the phase preference in FA and PA regions is caused by a positive activation to the –visible- peripheral condition. The same can be seen in the MD controls (Figure 8C and 8E), where the phase pattern in higher level areas is more similar to those of the JMD patient than in the lower visual areas. The activation patterns show that the same effects cause this phase sensitivity: positive activation in the peripheral condition, compared to negative or close to zero activations in the central and paracentral conditions (Figure 8D and 8F).

#### RP patient and controls

**Figure 9:**
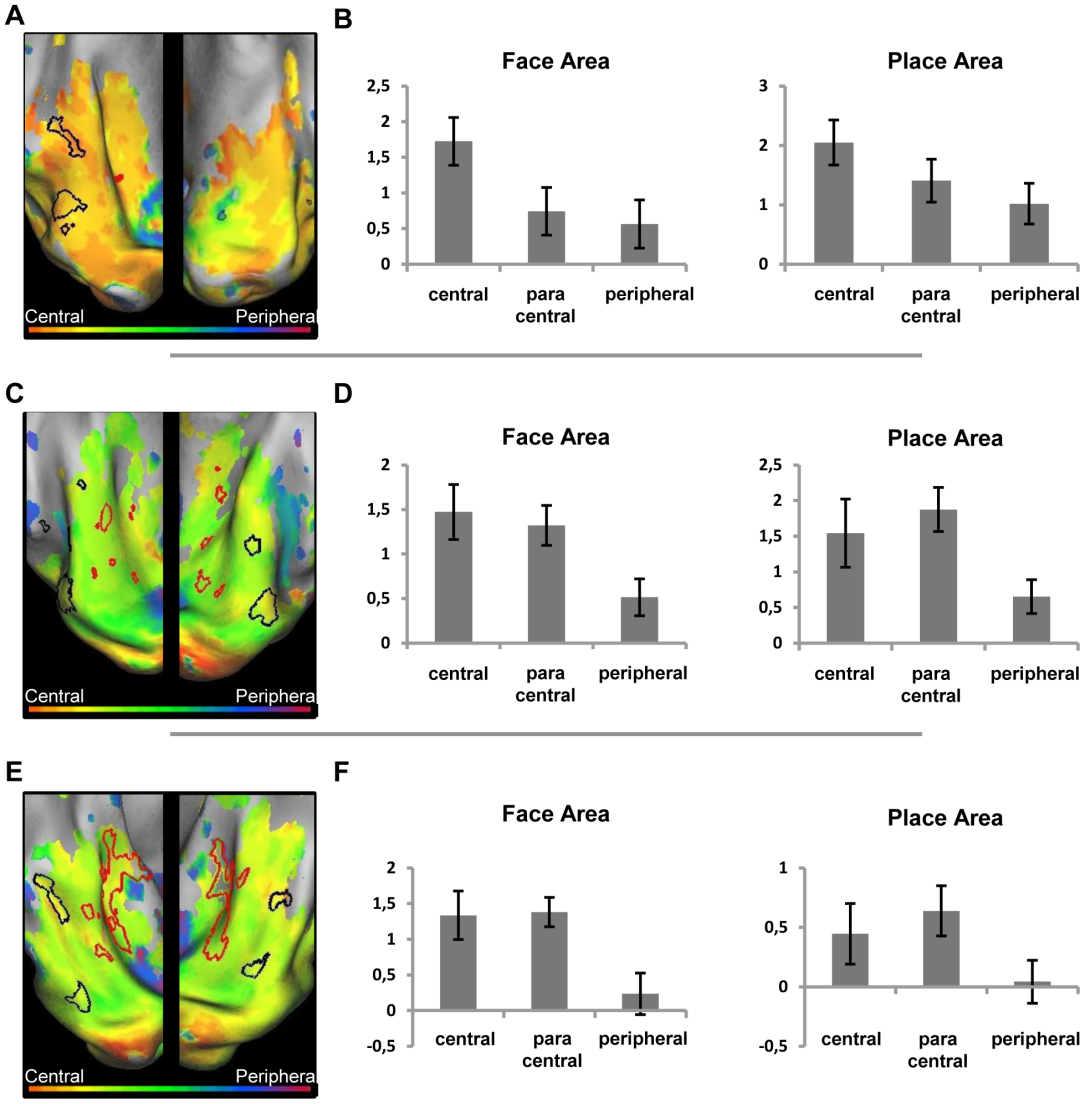
Preference and activity patterns for different eccentricities in the ventral cortex with peripheral (simulated) scotoma. (Left) Relative preference in the eccentricity mapping paradigm for the RP patient (A), control 1 (C) and control 2 (E), shown on an inflated hemisphere. The color legend is shown above (orange-red for central stimuli, green for paracentral stimuli, blue-purple for peripheral stimuli). The black lines mark the face-sensitive areas (FA), the red lines mark the house (place)-sensitive areas (PA) defined by the blocked localizer design. (Right) average beta values of three conditions, when the eccentricity data are analyzed as a block design and compared to a fixation baseline, in both the FA and PA region for the RP patient (B), control 1 (D) and control 2 (F).

Compared to the JMD patient, the opposite eccentricity pattern is found: the RP data show a strong preference for central stimuli (Figure 9A). Again, in this case the finding is not caused by deactivations (Figure 9B). Different from the JMD patient no deactivations can be seen in the less visible condition (peripheral). Most of the input which reaches the high level areas comes from central or paracentral stimuli. Consistent with the activation patterns in the calcarine sulcus, there are some responses present in the ventral occipitotemporal cortex to the peripheral stimuli, but they are weak compared to the (para)central activations. Again, the controls show a pattern that is very similar to the RP patient's data, in terms of phase preference (Figure 9C and 9E) as well as in terms of activation compared to baseline for the three conditions (Figure 9D and 9F): the eccentricity map in the object-selective areas FA and PA show a preference to (para)central stimuli, caused by a positive response in both areas to the visible stimuli, and a lower response to the less sensible peripheral stimuli.

The data in the occipitotemporal cortex were analyzed further by calculating the mean phase response for each ROI for both experimental conditions, in each hemisphere and for both patients (N = 3, with left/right FA for JMD and RP and right PA for JMD and RP; the fourth hemisphere is not included due to lack of left PA for RP). These mean phase responses illustrate the general preference of the ROI to the phase encoding stimuli. These stimuli, as explained above, correspond to values ranging on a scale from 0 to 2*pi. Values close to 0 represent face-like or central preferences, while values approximating 2*pi reveal preferences to house-like or peripherally presented stimuli. By comparing the mean preferences of these ROIs, the results of the previous section can be summarized and expanded. Table 1 shows the mean phase values for both subjects, in both hemispheres and for all ROIs, compared to the values of control 2 in a similar study under normal conditions (no simulated scotoma) (see [20]).

**Table 1:** Mean phase values per ROI for JMD, RP and control 2.

A		JMD	RP	C02
	Right FA	4.94	1.21	2.25
	Left FA	5.09	2.58	1.09
	Right PA	5.23	1.66	3.73
	Left PA	5.16	X	4.23

B		JMD	RP	C02
	Right FA	1.35	1.56	1.63
	Left FA	1.08	1.80	1.62
	Right PA	4.60	3.58	4.39
	Left PA	4.15	X	4.55

(A) The mean phase values in FA and PA per hemisphere for JMD and RP in the eccentricity mapping paradigm, and for control 2 in the eccentricity mapping paradigm with no simulated scotoma [20]. (B) The mean phase values in FA and PA per hemisphere for JMD and RP in the object-morph-paradigm, and for control 2 in the object-morph paradigm in a previous study [20] .

First, regarding the retinotopic data (Table 1A), the phase responses in the FA and PA are consistent with the patients' visible eccentricity: the mean phase preference across FA and PA in the JMD patient reveals a value that is consistent with a peripherally-oriented phase response (mean_JMD_  = 5.11), a similar value calculated for the RP patient reveals an opposite pattern, with a mean phase value corresponding to a centrally-oriented phase response (mean_RP_  = 1.82). This preference corresponds with the positive responses found in both FA and PA to the visible stimuli (central for RP, peripheral for JMD, see Figure 8 and 9). Additionally, a paired t-test which compared the mean phase values of FA and PA for both subjects revealed that these values found do not differ significantly (*p*_FA vs.PA_  = 0.13, df = 2), meaning that the phase responses found in FA and PA for each subject reflect the same stimulus preference. Another interesting finding is that when mean values are compared between the two patients, these do differ significantly (*p*_JMD vs.RP_  = 0.013, df = 2), so the mean preferences in the ventral visual stream for RP are different from those of JMD, and reflect a central vs. a peripheral bias, respectively. These results show that while JMD and RP show a different preference to different eccentricities, there is no difference in the response between FA and PA, which differs from results found in normal subjects where a dichotomy in eccentricity sensitivity in FA and PA can be found (FA more centrally oriented, PA more peripherally, see [20] and Table 1A). These results correspond with the properties of the pRF in object-selective cortex: The pRFs in those regions respond to more than one position in the visual field [11].

Combined with the JMD data, the activation patterns in the higher visual areas show that the visual field preferences in object-selective cortex differ from those in the lower visual areas. The preference in higher visual areas is very much dominated by the part of the visual field which is least affected by the retinal degeneration.

### Category sensitivity for faces and buildings

#### JMD patient, RP patient and control 2

**Figure 10:**
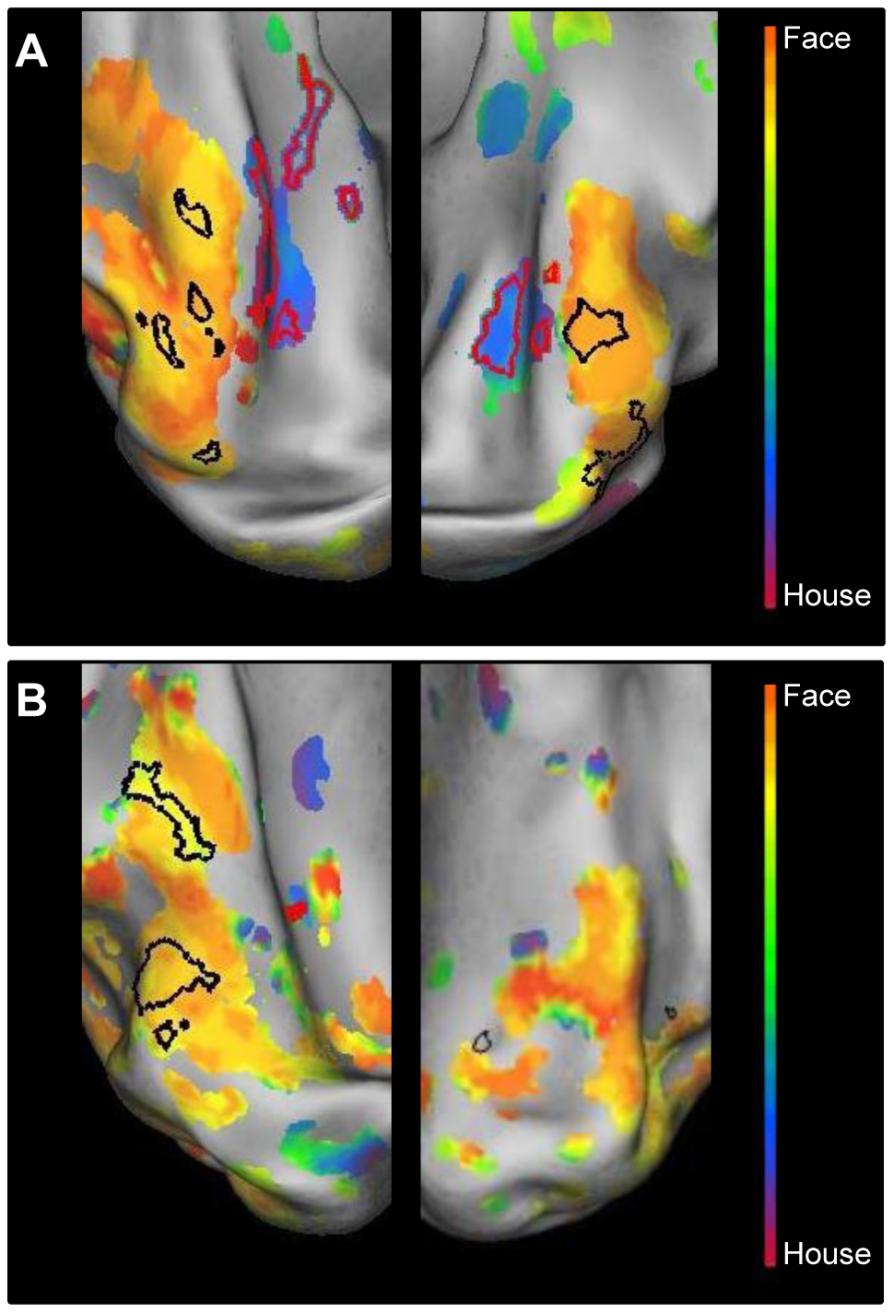
Relative preference to the object-morph stimuli in the ventral cortex of the two patients. Relative preference in the object-morph paradigm, shown on an inflated hemisphere for the JMD patient (A) and the RP patient (B). The color legend is shown above (orange-red for central stimuli, green for paracentral stimuli, blue-purple for peripheral stimuli). The black lines mark the face-sensitive areas; the red lines mark the house (place)-sensitive areas, defined by the blocked localizer design.

In Figure 10A and 10B, the results are shown for the phase-encoding responses to the object morph stimuli for the JMD (Figure 10A) and the RP patient (Figure 10B). The results of the localizer block data are marked, showing FA and PA. The face-sensitive and building-sensitive responses are in similar relative positions and of similar strength as seen in control subjects with normal vision (see e.g. Figure 8C and 8E for the position of FA and PA in controls; and [20]). Furthermore, as in control subjects, a correspondence is found between the face- and house-sensitive areas and the phase responses to the face and house-morph data, respectively. In the left ventral cortex, however, PA could not be defined for the RP patient, and the face-sensitive ROI is small. Nevertheless, this small face-sensitive region was close to a larger region with a face preference in the morphing experiment. Across both hemispheres, the house-sensitive responses seem smaller in the RP patient than in the JMD patient, and also smaller than in the subjects with normal vision tested in [20]. Aside from the phase-encoding and block data, the average beta value for these regions across conditions and across subjects (JMD, RP and Control 2) is positive (beta  = 0.92), demonstrating that responses in category-sensitive cortex are not the result of deactivations. When the data of the second control is left out, the average beta value is 0.91 across patients, indicating no abnormalities in responses for patients compared to a control.

As done previously for the eccentricity data, we also performed statistics on the phase responses to the morphed stimuli in ventral visual cortex. The results for the category-specific data (Table 1B) show a normal response in both FA and PA to faces and houses, respectively: mean phase responses in both patients' FA reveal a face-like preference (mean_FA_  = 1.45), while in PA we find a value that is clearly house-like in preference (mean_PA_  = 4.11). A t-test which explores the difference in phase preference between FA and PA for both subjects, shows that this difference is significant (*p*_FA vs.PA_  = 0.019, df = 2). When the mean phase responses of FA in JMD and RP, and PA in JMD and RP are compared, these values do not differ significantly (*p*_JMD vs.RP_  = 0.96, df = 2), which means that the preference patterns found in FA and PA are consistent across subjects. These results confirm patterns found in normal subjects, with phase responses corresponding to sensitivity to faces in FA and to houses in PA (See Table 1B and [20]).

In sum, while the relative invisibility of particular parts of the visual field in patients and in input-matched controls had a marked effect upon eccentricity maps in category-selective regions, the category sensitivity has been preserved: The category sensitivity in the current study for the patients and for input-matched controls was qualitatively very similar to the findings from an earlier study in controls tested with non-degraded visual stimulation [20].

## Discussion

In this study, we examined how retinal defects of the central and peripheral visual field influence eccentricity and category sensitivity mapping in visual cortex. In lower visual areas such as primary visual cortex, where voxels only respond to stimulation in a relatively small part of the visual field, many voxels are deprived of visual input when we map visual eccentricity. In such an absence of positive activation, negative BOLD responses (compared to a no-stimulus baseline) influence the phase-encoding pattern in lower visual areas. These distortions occur when the lack of activity in the lower cortical regions due to the (simulated) scotoma are compared with conditions where the same regions show a deactivation. These distortions can strongly influence the appearance of the eccentricity map compared with a control with normal vision (no real or simulated scotoma). The same problem does not occur in the object-morph paradigm: these stimuli were always visible and elicited category-sensitive activation spots that appeared to be normal and in line with category sensitivity in normal subjects. Nor does the problem occur for eccentricity mapping in high-level visual cortex where all voxels (known to have a large receptive field) show a preference for the most visible part of the visual field.

### Negative BOLD responses: General discussion

Negative BOLD responses (NBR) in unstimulated parts of the visual cortex are a widespread phenomenon. A number of studies with normal subjects have looked at NBR, its effect, size and possible origin [39], [40], [41]. These studies found that the NBR is robust and sustained, and mirrors the effect of the positive BOLD response (PBR). There is a lot of debate about the nature of the NBR, but a number of studies have found evidence for a neuronal origin of the NBR, meaning that the deactivation patterns are not purely caused by hemodynamic changes (‘blood stealing’), but are more likely due to a reduction or suppression of neural activity [39], [40], [41].

While the strength of the NBR appears to be on average lower than that of the PBR [40], [41], the deactivation is robust in that it is temporally and spatially highly reproducible. In addition, it is found clearly on a group level even if the threshold on an individual level needs to be lower to reveal the NBR. In our study, we used a low threshold to look at the NBR, indicating that indeed the effects are not as strong as for the PBR. Nevertheless, the different thresholds used in the Supporting Information show that in some cases, NBRs are strong and prevalent even at a high threshold (p<0.0001 uncorrected, see e.g. Figure S2B and S4F). In the JMD patient, the NBR is very strong, and affects the retinotopic map at higher thresholds than p<0.05 uncorrected. In some other subjects, the NBRs are smaller, but can be reproduced. In particular, the RP patient performed the same eccentricity mapping paradigm with a slightly different task, and a very similar pattern of NBRs can be found (see Supporting Information, compare Figure S1 with main Figure 6). Control 2 does not show very strong NBRs in the JMD condition which is characterized by very little visual input (see Supporting Information, Figure S3B), but has strong deactivations in the two conditions with more input: when no simulated scotoma is present and in the RP condition (see Supporting Information, Figure S6 and Figure S4F). This demonstrates that while NBRs might seem to have only a limited impact, they are a widespread phenomenon and they could influence results more than initially suspected. The size of this influence is likely different between subjects given the large interindividual variability in the strength of NBRs. This makes it even more important to investigate the presence of NBRs when investigating individual patients.

### Deactivations and null responses: A further discussion

NBRs have been reported in studies which investigated retinotopic organization in MD as well as RP [30], [31], [32], [33]. When different stimulus conditions were compared with each other rather than with a no-stimulus baseline, such NBRs will go unnoticed and might distort the retinotopic maps, as demonstrated by our current results. This is not a trivial finding, and it is a crucial point to take into account when analyzing fMRI data and interpreting findings in the literature.

For example, [42] mapped retinotopic organization in MD subjects as well as controls with a simulated central scotoma. No mean-luminance (no-stimulus) baseline periods were inserted in the presentation of their retinotopic mapping stimuli. It is therefore possible that deactivation patterns would contribute to some of the results. For instance, based on the eccentricity mapping data, population receptive fields (pRFs) were modeled for voxels inside the LPZ of pathological and simulated central lesions. For both types of lesions, the LPZ exhibited ectopic pRFs, i.e. pRFs shifted from their normal central visual field position towards paracentral positions. These ectopic pRFs were explained by LPZ neurons with large and more eccentric RFs, having a larger weight on the voxel's response in the absence of central stimulation. However, an alternative account is possible as well: If LPZ voxels would become deactivated by the more peripheral ring stimuli, while other eccentricity stimuli elicit neither a negative nor a positive response, then the no-response for paracentral stimuli will come out as stronger than the negative response for peripheral stimuli and a paracentral preference could be inferred (although no genuine positive response was present).

Conceivably, NBRs could contribute differentially to the results of patients and controls, perhaps masking a possible difference between both subject groups. This has been suggested by [33], where differences in task leads to differences in response patterns (positive as well as negative) between RP patients and controls. Our data, when looking at the strength of the NBR responses (Supporting Information, Figures S2 and S4) also suggest that strength of NBRs can differ between patients and controls. In conclusion, while we do not wish to invalidate the results of [42], the possibility remains that this alternative explanation might mask some of their results, indicating the need of clear comparisons with baseline measures in all studies where lack of input might be influenced by NBRs. Our study only investigated eccentricity mapping. Another aspect of retinotopic mapping is polar angle. It would be interesting to investigate if and how polar angle is affected by loss of input, and it might provide additional information about how retinotopy is affected by visual loss. However, we think it is prudent that baseline comparisons are always included in the analysis, as it is the most fool proof way to rule out artefacts caused by NBRs.

The potential danger related to deactivation patterns is not confined to retinotopic mapping in subjects with a loss of input due to visual abnormalities. Note that in a conventional phase-encoding retinotopic mapping procedure of normally-sighted subjects typically a large part of the peripheral visual field remains unstimulated, given the limited size of the projection screen. This can be regarded as a loss of input as well. Consequently, part of anterior occipital cortex is not activated but could nonetheless become deactivated by more centrally located stimuli. The second control in this study, also participated in a previous experiment where no scotoma was simulated, and there NBRs can be seen in every unstimulated part of the calcarine sulcus (Supporting Information, S6). When applying a Fourier analysis without comparisons to a zero-contrast baseline, the effect of negative responses to central stimuli compared to zero responses to peripheral stimuli would yield a disproportionately large preference to peripheral stimuli in the anterior parts of the calcarine sulcus, not corresponding to the actual amount of peripheral stimulation. Given that quite a few studies that use phase-encoding in the literature did not report using a no-stimulus baseline, the phenomenon that we observed here very strongly in a rather specific case study might have had an influence on the findings in many publications. While these effects would not normally have a large effect in phase-encoding studies with normal subjects (only affecting the amount of peripheral preference, as stated above), in patient studies it is very important to ensure that the analyses used to explore the data correctly represent the actual activation to the displayed stimuli.

### Eccentricity mapping and category sensitivity in more high-level regions

The category-sensitive regions in the ventral visual stream are not affected by NBRs in the same way as the lower visual regions. Any deactivations occurring in these areas are only present in conditions where no visual input is present, and they are countered by a positive response to visible stimuli. In the patients, input in the ventral visual stream is limited to either central (RP) or peripheral stimuli (JMD), and thus no (weak) retinotopic map is found in our eccentricity mapping. While under normal viewing conditions a preference is found for central stimuli in FA and for peripheral stimuli in PA, this pattern disappears and is replaced with a general preference to one type of stimuli, depending on the input, central (RP) or peripheral (JMD). This response pattern can be explained in terms of pRF properties [11]. While the lower visual areas have small pRF sizes and respond only to a fraction of the visual field, the voxels in object-selective cortex have larger pRF sizes and they respond to a wider range of visual input from the visual field.

The lack of retinotopic organization caused by lack of visual input to certain eccentricities do not affect the object-sensitive preferences of higher visual areas: the object-morph paradigm results in a normal preference to face-like stimuli in FAs and house-like stimuli in PAs of both patients, similar to those found in controls. An investigation of the response patterns shows mostly positive activation to all object-stimuli in these regions. This stands in apparent contrast with a reduction of activity in face-sensitive or object-selective areas following developmental amblyopia [43] or restoration of vision after blindness [44], [45]. A face-sensitive deficit was assumed to be related to a selective abnormality of central visual field processing in [43], while the results of [44] might arise from a failure to develop V1 neurons with small receptive fields. Additionally, another study investigates a patient group where the fusiform face area (FFA) is enlarged, rather than less responsive [46], indicating that some plasticity might exist concerning the object-selective areas. The JMD patient did develop face-related areas despite the significant loss of vision, but her visual deprivation was not as pronounced as amblyopic deficits or blindness in the first years of life. Complementary to the findings in central vision defects leading to reduced face-sensitive activity, one can assume a similar process might occur in peripheral vision loss with the place-sensitive activity. The RP subject did show a reduced or non-existent PA. However, without a larger sample of patients and a proper control group it is not possible to draw any conclusions from this finding. Moreover, it was recently shown that a parahippocampal place area (PPA) can be established using auditory stimulation not only in normally sighted but also in congenitally blind subjects [47], suggesting that visual experience is not required to develop category sensitivity in PPA.

### Cortical reorganization and plasticity after retinal deprivation

Our study does not give any indication for major cortical reorganization as a consequence of the retinal deprivation: the combination of lack of activation due to the retinal lesions and deactivation in the corresponding parts of the visual cortex fully explain our results. While the study was not designed to look specifically at plasticity issues, it remains interesting to examine if and how these results contribute to a recent controversy in the literature. Some contradictory results have been found regarding plasticity in MD patients: [42] and [48] did not find any alterations in the responsiveness of the LPZ, contrary to the results of Baker and Dilks and their colleagues who did report significant activation in the LPZ [30], [31], [49].This difference has been attributed to differences in task demands: A stimulus-related task would elicit responses in the LPZ, and a stimulus-unrelated task or passive viewing would not [32]. A similar phenomenon would be operational in RP patients as well [33]. Our study did not specifically manipulate task demands to investigate this claim. The subjects were asked to detect the stimulus with the lowest luminance, a task which could be classified as a stimulus-related task. However, the task could be completed successfully by using the luminance level of the stimuli as a cue without paying attention to the actual content of the depicted images, so its exact classification is debatable. The RP patient was asked to respond to luminance changes in the fixation point in a different version of the study, a clear stimulus-unrelated task (see Supporting Figure S1). In both tasks, the same lack of reorganization and appearance of NBR could be found.

Additionally, the results of the control data do not contribute to the question of possible reorganization in the lower visual areas of JMD and RP patients either. In the RP patient, phase-encoding patterns where very similar between RP and controls, while the JMD patient and its controls show different phase preferences. When looking at the response patterns compared to baseline, at different thresholds (see Supporting Figure S2 and S4), there is not much consistency in the strength of the responses of patients compared to controls. The JMD controls tend to show weaker NBR than the JMD patient, while the RP patient has one control with much stronger NBR, and one with a more similar response pattern. Our study contains enough subjects to demonstrate that NBR has to be taken into account for retinotopic mapping, and that the strength of NBR varies between subjects, but not to make any claims about cortical plasticity nor to suggest any difference between patients and controls in the presence and strength of NBR.

Masuda and colleagues [33] do show a difference in RP patients and their controls in responding to task demands, with less deactivation in stimulus-related judgments than in a passive viewing task. In our study, we see slight differences in response strength for the NBR between the RP patient and his controls, but no clear differences in PBR. Another study by Masuda and colleagues [32] shows similar effects in the JMD patients as for their RP patients study, but our findings show that the NBR in the JMD patient was much stronger than for its controls. The role of attention and task demands becomes even more complicated when taking into account another study [50], where it is suggested that attention focused on a stimulus might lead to higher suppression of activity in other parts of the lower visual cortex, suggesting more deactivation in stimulus-related tasks than in passive viewing tasks under normal viewing conditions. Given these data, the use of a fixation task might cause differences in activation patterns depending on whether the stimuli presented are central (around fixation) or more peripheral (away from fixation).

The above complications in testing for task demands and attention effects fall outside the scope of what we set out to investigate in our study. While we did not manipulate task demands in order to test such effects, our findings are consistent with the proposal that cortical reorganization, if it exists, is not a universal phenomenon occurring under all task conditions.

In conclusion, the current study highlights the importance of distinguishing between activity maps and preference maps in the investigation of retinotopic maps and visual field preferences, and provide a first bench mark for future studies on the effect of retinal defects on the functional organization in high level visual regions.

## Supporting Information

Figure S1 **RP results of the eccentricity mapping paradigm with a fixation dimming task.** The RP patient completed 12 runs of the same eccentricity experiment, where the task was to respond to a reduction in the luminance of the fixation spot. (A) Relative preference for the RP patient in the eccentricity mapping paradigm. An inflated medial view of the right and left hemisphere is shown. (B) The response pattern of the RP patient in three conditions when the data are analyzed as a block design, compared with a baseline condition, at p<0.05 uncorrected (central: 8 most central stimuli; paracentral: 8 middle stimuli; peripheral: 8 most eccentric stimuli.
(TIF)

Figure S2 **Activity patterns for the JMD patient and controls at different thresholds.** (Left) Activity patterns for the three conditions of the block design at p<0.01 uncorrected for the JMD patient (A), control 1 (C) and control 2 (E). (Right) Activity patterns for the three conditions of the block design at p<0.0001 uncorrected for the JMD patient (B), control 1 (D) and control 2 (F).
(TIF)

Figure S3 **Activity patterns for the two controls with a JMD simulated scotoma.** Data for control 1 (A) and control 2 (B), when comparing three conditions with a fixation baseline at p<0.05 uncorrected (central: 8 most central stimuli; paracentral: 8 middle stimuli; peripheral: 8 most eccentric stimuli). For each control, two regions are marked in black which are further characterized for illustration purposes. They represent the effects of positive activations and negative activations compared to a fixation baseline.
(TIF)

Figure S4 **Activity patterns for the RP patient and controls at different thresholds.** (Left) Activity patterns for the three conditions of the block design at p<0.01 uncorrected for the RP patient (A), control 1 (C) and control 2 (E). (Right) Activity patterns for the three conditions of the block design at p<0.0001 uncorrected for the RP patient (B), control 1 (D) and control 2 (F).
(TIF)

Figure S5 **Activity patterns for the two controls with a RP simulated scotoma.** Data for control 1 (A) and control 2 (B), when comparing three conditions with a fixation baseline at p<0.05 uncorrected (central: 8 most central stimuli; paracentral: 8 middle stimuli; peripheral: 8 most eccentric stimuli). For each control, two regions are marked in black which are further characterized for illustration purposes. They represent the effects of positive activations and negative activations compared to a fixation baseline.
(TIF)

Figure S6 **Activity patterns for control 2 in a normal eccentricity mapping paradigm (no simulated scotoma).** The response pattern of control 2 in three conditions when the data are analyzed as a block design, compared with a baseline condition, at p<0.05 uncorrected (central: 8 most central stimuli; paracentral: 8 middle stimuli; peripheral: 8 most eccentric stimuli. The data were collected in a previous experiment [20].
(TIF)
