# Supplementary figures and images for: Visual Space and Object Space in the Cerebral Cortex of Retinal Disease Patients
Source: PLoS One. 2014 Feb 5;9(2):e88248. doi: 10.1371/journal.pone.0088248 (PMC3914958; doi:10.1371/journal.pone.0088248)

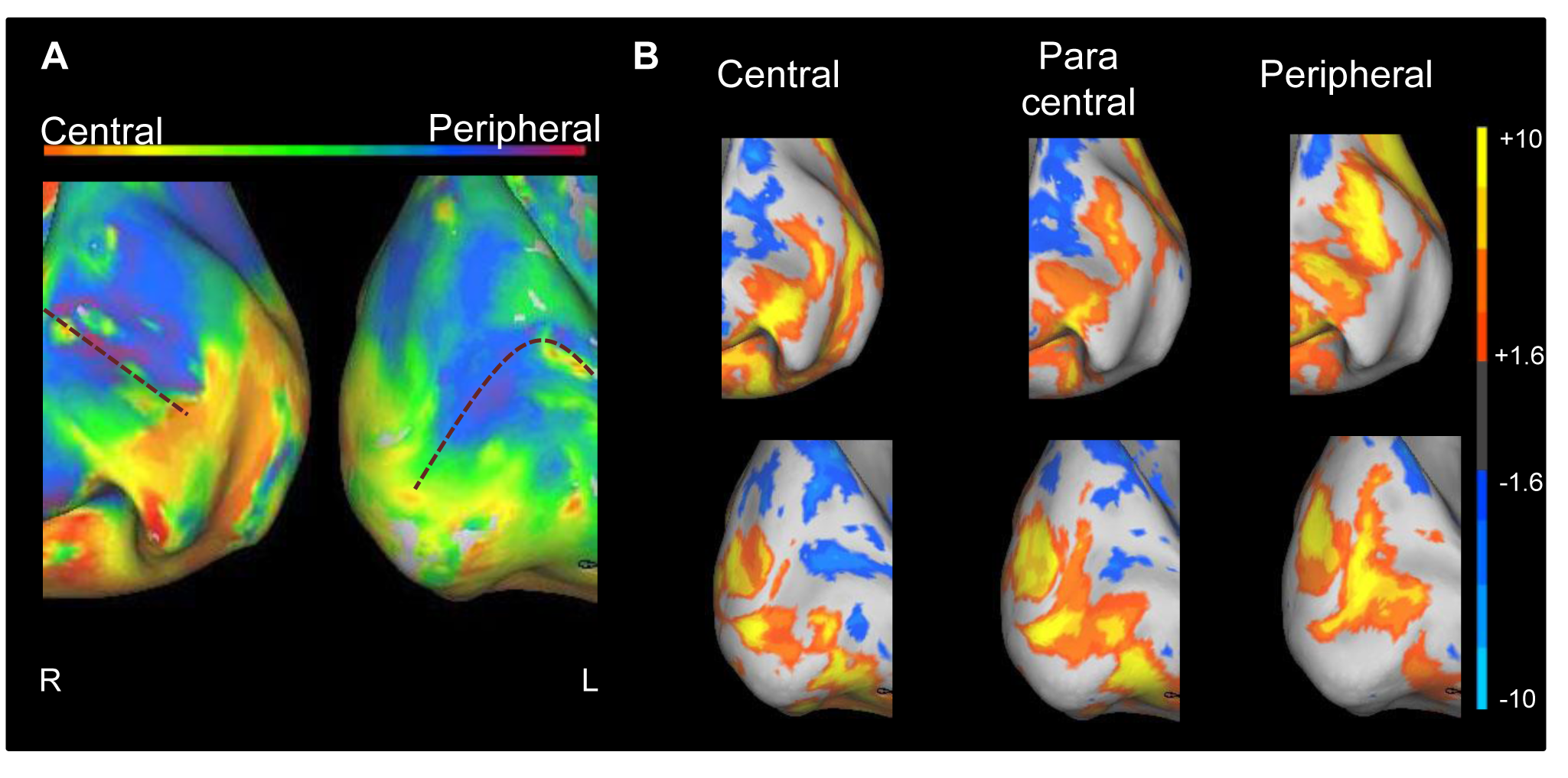

Supplement: Figure S1 — RP results of the eccentricity mapping paradigm with a fixation dimming task. The RP patient completed 12 runs of the same eccentricity experiment, where the task was to respond to a reduction in the luminance of the fixation spot. (A) Relative preference for the RP patient in the eccentricity mapping paradigm. An inflated medial view of the right and left hemisphere is shown. (B) The response pattern of the RP patient in three conditions when the data are analyzed as a block design, compared with a baseline condition, at p<0.05 uncorrected (central: 8 most central stimuli; paracentral: 8 middle stimuli; peripheral: 8 most eccentric stimuli. (TIF) [file pone.0088248.s001.tif]

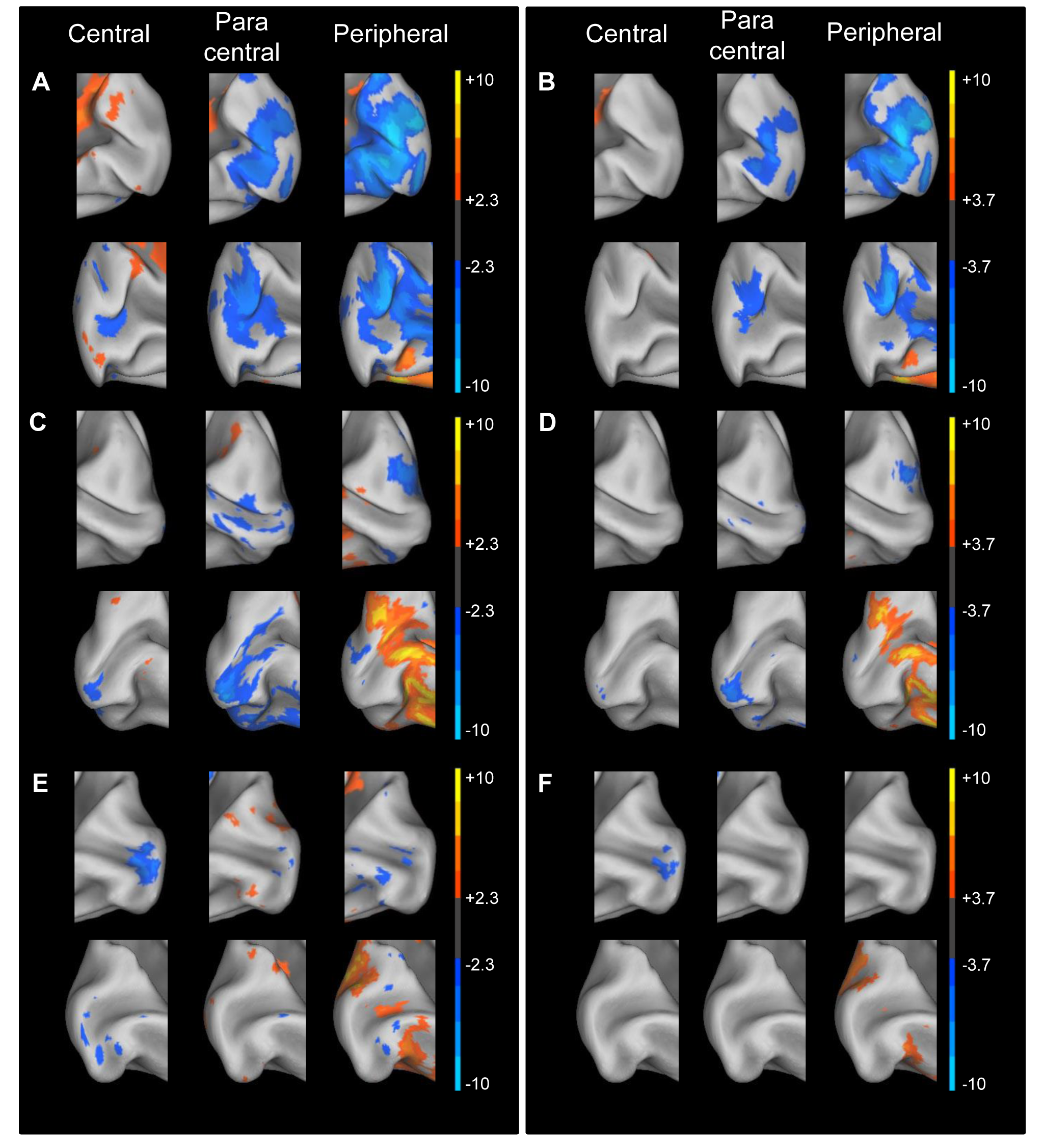

Supplement: Figure S2 — Activity patterns for the JMD patient and controls at different thresholds. (Left) Activity patterns for the three conditions of the block design at p<0.01 uncorrected for the JMD patient (A), control 1 (C) and control 2 (E). (Right) Activity patterns for the three conditions of the block design at p<0.0001 uncorrected for the JMD patient (B), control 1 (D) and control 2 (F). (TIF) [file pone.0088248.s002.tif]

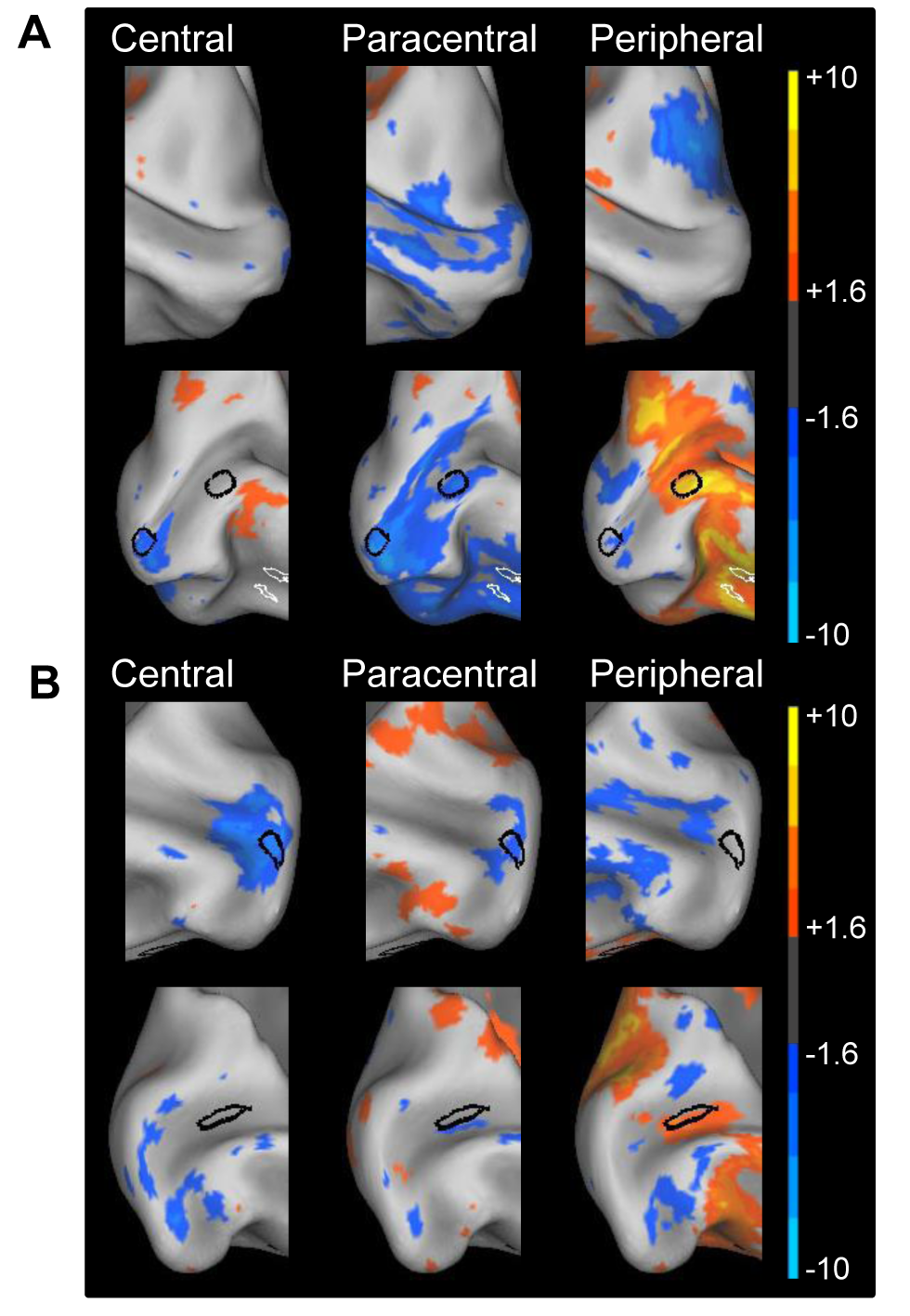

Supplement: Figure S3 — Activity patterns for the two controls with a JMD simulated scotoma. Data for control 1 (A) and control 2 (B), when comparing three conditions with a fixation baseline at p<0.05 uncorrected (central: 8 most central stimuli; paracentral: 8 middle stimuli; peripheral: 8 most eccentric stimuli). For each control, two regions are marked in black which are further characterized for illustration purposes. They represent the effects of positive activations and negative activations compared to a fixation baseline. (TIF) [file pone.0088248.s003.tif]

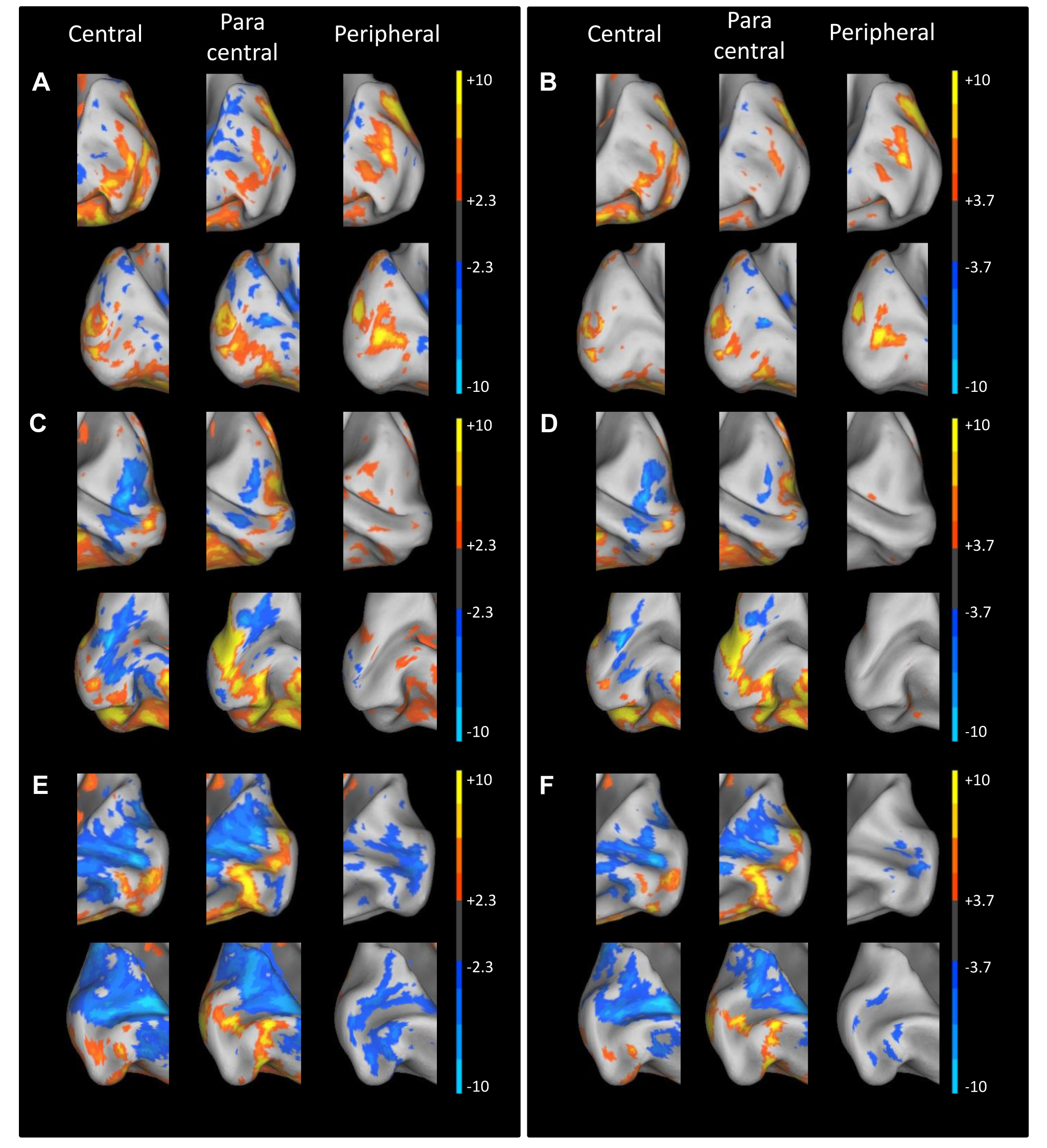

Supplement: Figure S4 — Activity patterns for the RP patient and controls at different thresholds. (Left) Activity patterns for the three conditions of the block design at p<0.01 uncorrected for the RP patient (A), control 1 (C) and control 2 (E). (Right) Activity patterns for the three conditions of the block design at p<0.0001 uncorrected for the RP patient (B), control 1 (D) and control 2 (F). (TIF) [file pone.0088248.s004.tif]

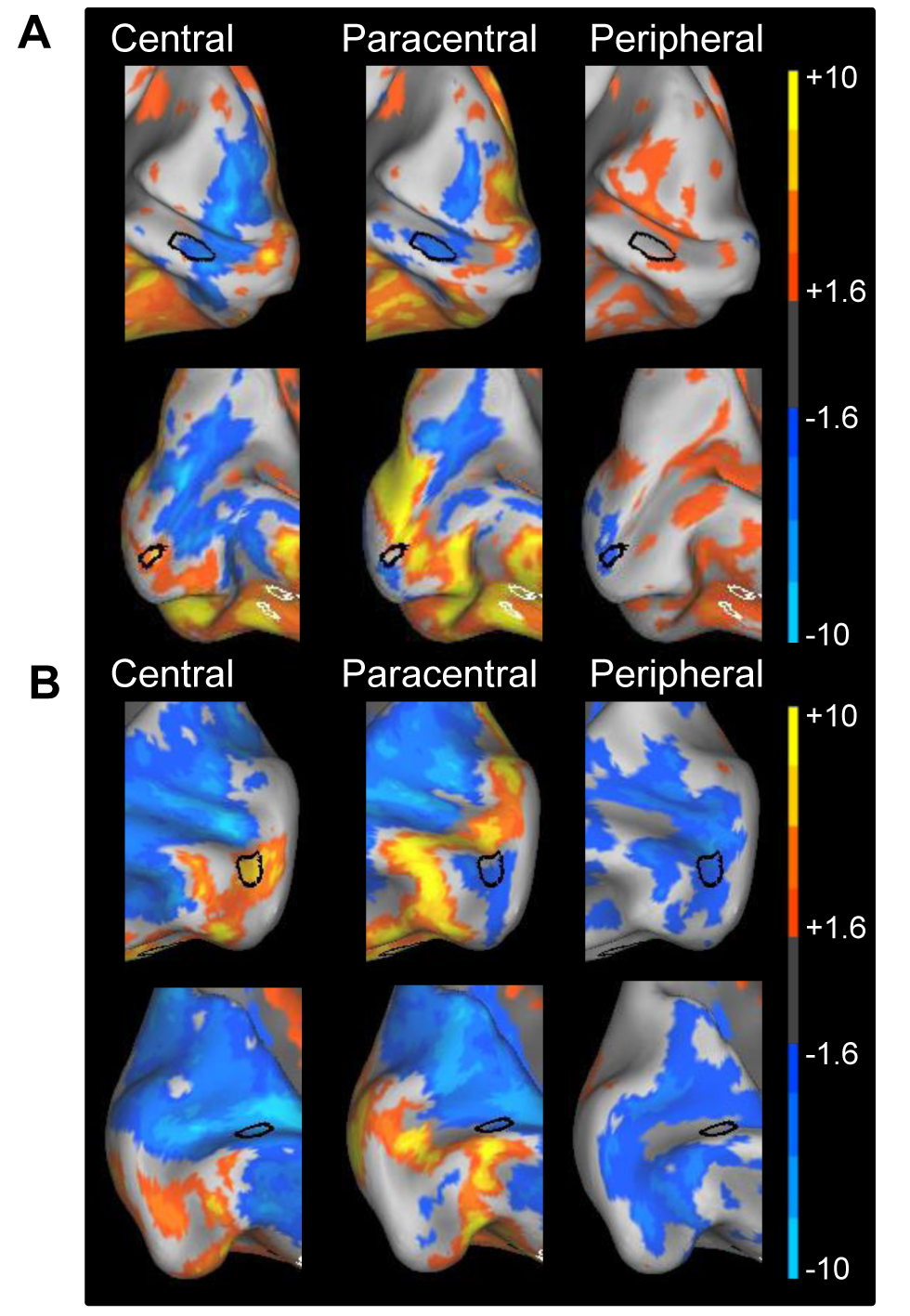

Supplement: Figure S5 — Activity patterns for the two controls with a RP simulated scotoma. Data for control 1 (A) and control 2 (B), when comparing three conditions with a fixation baseline at p<0.05 uncorrected (central: 8 most central stimuli; paracentral: 8 middle stimuli; peripheral: 8 most eccentric stimuli). For each control, two regions are marked in black which are further characterized for illustration purposes. They represent the effects of positive activations and negative activations compared to a fixation baseline. (TIF) [file pone.0088248.s005.tif]

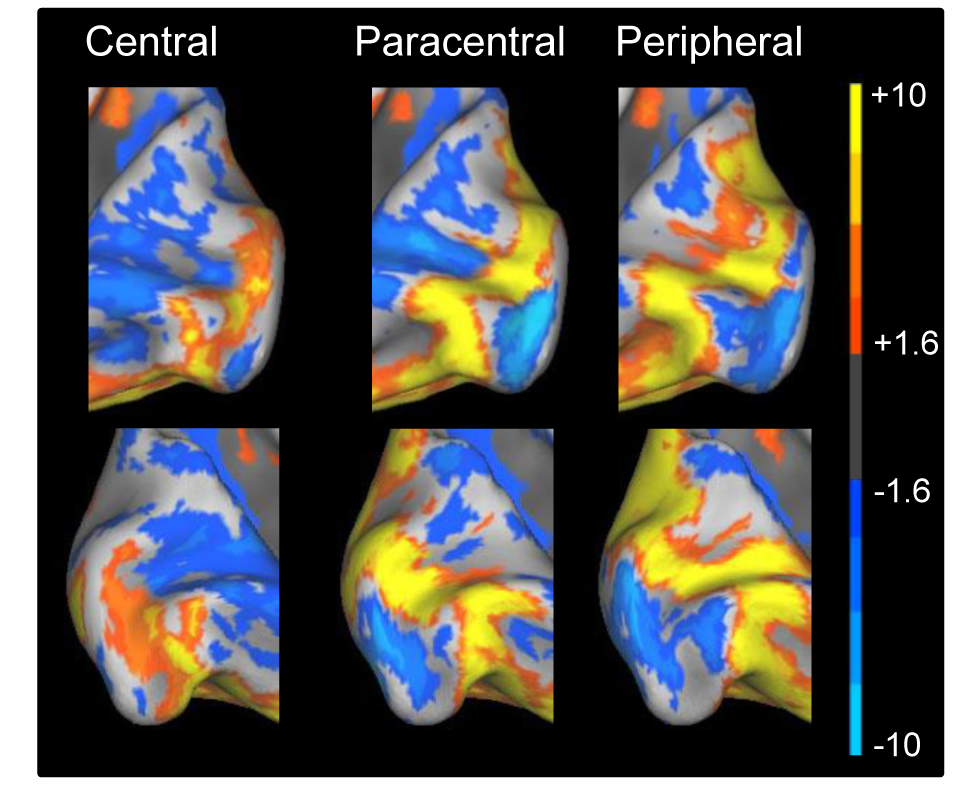

Supplement: Figure S6 — Activity patterns for control 2 in a normal eccentricity mapping paradigm (no simulated scotoma). The response pattern of control 2 in three conditions when the data are analyzed as a block design, compared with a baseline condition, at p<0.05 uncorrected (central: 8 most central stimuli; paracentral: 8 middle stimuli; peripheral: 8 most eccentric stimuli. The data were collected in a previous experiment [20]. (TIF) [file pone.0088248.s006.tif]
